# Maprotiline restores ER homeostasis and rescues neurodegeneration via Histamine Receptor H1 inhibition in retinal ganglion cells

**DOI:** 10.1038/s41467-022-34682-y

**Published:** 2022-11-10

**Authors:** Wei Chen, Pingting Liu, Dong Liu, Haoliang Huang, Xue Feng, Fang Fang, Liang Li, Jian Wu, Liang Liu, David E. Solow-Cordero, Yang Hu

**Affiliations:** 1 Department of Ophthalmology, Stanford University School of Medicine, Palo Alto, CA 94304 USA; 2 High-Throughput Bioscience Center, Stanford University School of Medicine, Palo Alto, CA 94305 USA; 3 Present Address: Multiscale Research Institute of Complex Systems, Fudan University, Shanghai, 201203 China; 4 Present Address: Department of Ophthalmology, The Second Xiangya Hospital, Central South University, Changsha, 410011 China; 5 Present Address: Beijing Institute of Ophthalmology, Beijing Tongren Eye Center, Beijing Tongren Hospital, Capital Medical University, Beijing, 100730 China

**Keywords:** Optic nerve diseases, High-throughput screening

## Abstract

When the protein or calcium homeostasis of the endoplasmic reticulum (ER) is adversely altered, cells experience ER stress that leads to various diseases including neurodegeneration. Genetic deletion of an ER stress downstream effector, CHOP, significantly protects neuron somata and axons. Here we report that three tricyclic compounds identified through a small-scale high throughput screening using a CHOP promoter-driven luciferase cell-based assay, effectively inhibit ER stress by antagonizing their common target, histamine receptor H1 (HRH1). We further demonstrated that systemic administration of one of these compounds, maprotiline, or CRISPR-mediated retinal ganglion cell (RGC)-specific HRH1 inhibition, delivers considerable neuroprotection of both RGC somata and axons and preservation of visual function in two mouse optic neuropathy models. Finally, we determine that maprotiline restores ER homeostasis by inhibiting HRH1-mediated Ca^2+^ release from ER. In this work we establish maprotiline as a candidate neuroprotectant and HRH1 as a potential therapeutic target for glaucoma.

## Abstract (web summary)

ER stress is associated with various neurodegenerative diseases. This study found that FDA approved drug, maprotiline, inhibits histamine receptor H1-mediated ER stress and provides significant neuroprotection in mouse glaucoma model.

## Introduction

The most common cause of irreversible blindness, glaucoma, will affect an estimated 3% of the world population over 40 years old by 2040 (more than 100 million people)^1^, which will impose a multi-billion dollar economic burden on society^2^. Glaucoma is characterized by optic neuropathy with optic nerve (ON) degeneration followed by progressive retinal ganglion cell (RGC) death^3–7^. The only available treatments act by reducing intraocular pressure (IOP), a risk factor associated with glaucoma^8,9^. However, IOP reduction fails to completely prevent the progression of glaucomatous neurodegeneration, indicating the urgent need for innovative neuroprotection therapies^10–13^.

We previously found that ON injury induces neuronal endoplasmic reticulum (ER) stress in RGCs^14^, suggesting a detrimental role of RGC-specific ER stress in glaucoma^15,16^. When the protein or calcium homeostasis of the ER is adversely altered, cells experience ER stress and activate three signaling pathways initiated by three ER-resident stress-sensing proteins: inositol-requiring protein-1 (IRE1α), activating transcription factor-6 (ATF6) and protein kinase RNA-like ER kinase (PERK), together called the unfolded protein response (UPR)^17,18^. IRE1α, a bi-functional enzyme that contains both a Ser/Thr kinase domain and an endoribonuclease (RNase) domain, mediates the splicing of X-box binding protein 1 (XBP-1) mRNA to generate an active (spliced) form of the transcription factor, XBP-1s. The IRE1α-XBP-1s pathway targets genes that increase ER protein-folding capacity and facilitate degradation of misfolded proteins. On the other hand, IRE1α kinase activity also activates pro-apoptotic c-Jun kinase (JNK), which contributes to Bax-dependent IRE1α-induced apoptosis^19^. ATF6 is a transcription factor that is truncated and thereby activated by ER stress to control the expression of a group of UPR target genes. PERK phosphorylates and inactivates eukaryotic translation initiation factor 2α (eIF2α) to attenuate global cap-dependent mRNA translation and thereby reduce protein load on the ER. Activating transcription factor 4 (ATF4) downstream of PERK-eIF2α induces expression of ER stress-specific transcription factor C/EBP homologous protein (CHOP)^20^. CHOP is a well-known pro-apoptotic transcription factor that mediates ER stress-induced cell death by downregulating anti-apoptotic Bcl2, upregulating pro-apoptotic BH-3 only molecules Bim and PUMA, increasing expression of death receptor 5 (DR5) and caspase 8 cleavage^16^. ATF4 can also form heterodimers with CHOP to cause cell death by upregulating protein synthesis and inducing oxidative stress^21^. Chronic ER stress with prolonged PERK-eIF2α-ATF4-CHOP signaling has been associated with many acute and chronic neurodegenerative diseases; genetic manipulation and small molecular modulators of this pathway have proven to be beneficial in animal models of various neurodegenerative diseases^17,18,22^.

We also previously found that genetic inhibition of CHOP or its upstream regulator eIF2α significantly protects RGCs’ somata and axons and preserves visual functions in mouse models of traumatic ON injury, glaucoma, and optic neuritis^14,23–25^. Therefore, identification of small modulators of ER stress to block CHOP is an important step toward developing effective neuroprotectants for glaucoma. We reasoned that identifying CHOP inhibitors from FDA-approved drugs would significantly shorten the drug development process. Therefore, we used a reporter cell line expressing CHOP promoter-driven luciferase to perform a high throughput screening (HTS) of five compound libraries with a total of 4846 compounds that have known bioactivities and have been approved for clinical application.

In this work, we identify three FDA-approved drugs, amoxapine, desloratadine, and maprotiline, as potent ER stress inhibitors. They share similar tricyclic chemical structures and a common antagonistic target, histamine receptor H1 (HRH1), through which we find that they restore ER homeostasis and achieve significant neuroprotection of RGCs and ONs in vivo in two mouse optic neuropathy models. Finally, we determine that maprotiline inhibits HRH1-mediated ER Ca^2+^ release and thereby inhibits the damaging intracellular Ca^2+^ influx induced by axon injury. This readily testable small molecule drug is a promising neuroprotectant, and HRH1 is a potential therapeutic target for glaucoma and other neurodegenerative diseases associated with ER stress.

## Results

### CHOP pathway inhibitors identified through cell-based HTS of FDA-approved drug libraries

**Fig. 1:**
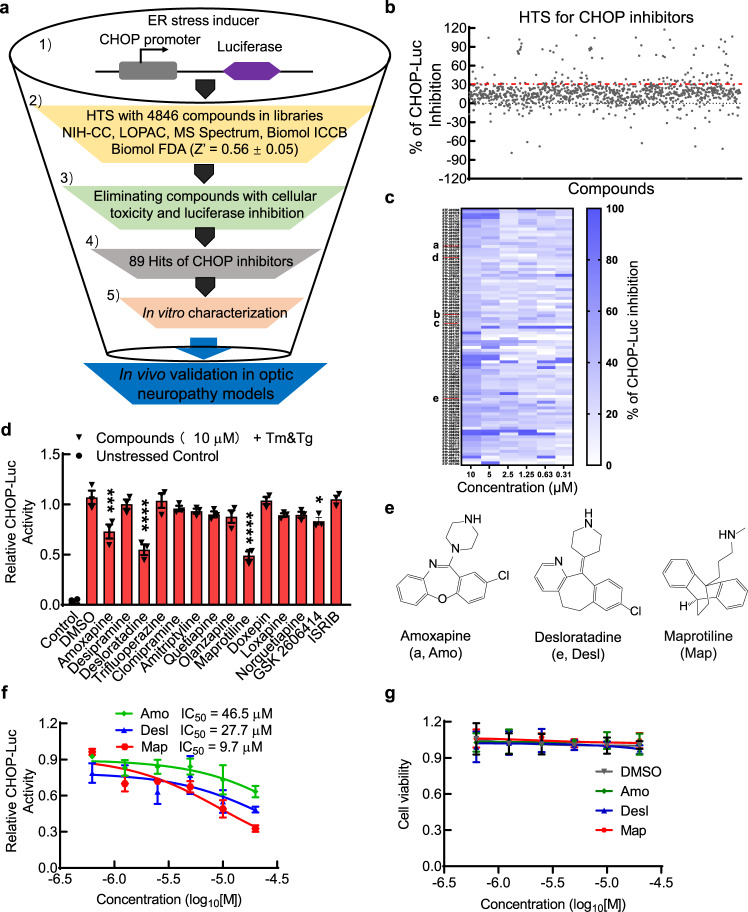
Cell-based HTS to identify small molecule inhibitors of the CHOP branch of UPR. **a** The schematic HTS pipeline employed to identify small molecules that inhibit the CHOP pathway of the ER stress with the HEK293T reporter cell line expressing CHOP promoter-driven luciferase (CHOP-Luc) and downstream in vitro and in vivo assays for further characterization. **b** Plot showing the percentage inhibition of CHOP-Luc signals of each tested library compound in the presence of Tm/Tg (1 µM), 24 h after exposure. “Hit” threshold is set at >30% inhibition (red dotted line) but <100% inhibition. **c** Heatmap of dose-dependent responses of 89 “hits” in CHOP-Luc inhibition. Compounds a–e with similar chemical structure are marked with red lines. **d** Relative (to DMSO) CHOP-Luc activities of 12 compounds and 2 control compounds GSK2606414 and ISRIB at 10 µM in the presence of Tm/Tg (1 µM), 24 h after exposure. Data are presented as means ± s.e.m., *n* = 3 independent replicates, ****P < 0.0001, ***P < 0.001, *P < 0.05, one-way ANOVA with Dunnett’s multiple comparisons test. **e** Chemical structures of amoxapine (Amo), desloratadine (Desl), and maprotiline (Map). **f** IC_50_ calculated with nonlinear regression through dose-dependent fits of CHOP-Luc activities (relative to DMSO) of individual compounds at indicated concentration in the presence of Tm/Tg (1 µM), 24 h after exposure. Data are presented as means ± s.e.m., *n* = 3 independent replicates. **g** Cell viability assay of Amo, Desl, and Map on HEK293T cells. Data are presented as means ± s.e.m., *n* = 3 independent replicates. Source data are provided as a Source data file.

We generated a stable cell line to express CHOP promoter-driven luciferase (CHOP-Luc) in HEK293T cells. This promoter shows dose-dependent responses to the ER stress inducers thapsigargin (Tg) and tunicamycin (Tm) (Supplementary Fig. 1a–c). We used this reporter cell line in a multiple dose-response assay to screen 4846 compounds from five FDA-approved drug libraries. All compounds were run in 7-point dose response based on a previous publication^26^, except the NIH Clinical Collection (446 compounds), which were tested in duplicate due to the small quantity of compounds on hand. We identified 89 “hits” (Fig. 1a–c) based on the criteria: (1) >30% inhibition of Tm/Tg induced CHOP-Luc signal; (2) no cell toxicity or luciferase inhibition activity; (3) dose-dependent effect. Among the 89 hit compounds, five (a–e) with good dose-dependent inhibition of CHOP-Luc share a similar tricyclic chemical structure (Supplementary Fig. 1d). We then focused on this series of compounds and added nine more tricyclic or tetracyclic FDA-approved small molecule drugs (not in the libraries) for retesting with the CHOP-Luc reporter line; we excluded compounds “c” and “d” because they are not commercially available (Supplementary Fig. 1d). Among the 12 retested compounds, only three (amoxapine, desloratadine, and maprotiline) significantly inhibited CHOP-luciferase activity induced by ER stress (Tm/Tg) at 10 µM (Fig. 1d). Two known inhibitors of the PERK-CHOP pathway, GSK2606414 and ISRIB^27^, were used as reference compounds. However, only GSK2606414 but not ISRIB significantly inhibited CHOP-luciferase activity induced by Tm/Tg (Fig. 1d and Supplementary Fig. 1e), indicating the low sensitivity of the reporter line. Another five compounds (desipramine, trifluoperazine, clomipramine, amitriptyline, olanzapine) inhibited CHOP-luciferase activity at 20 µM (Supplementary Fig. 1e). Interestingly, a previous study also identified trifluoperazine as a CHOP inhibitor using a different CHO luciferase reporter cell line driven by murine CHOP promoter^28^. Maprotiline appeared to be the most potent of the three as it has the lowest IC_50_ (Fig. 1e, f). Importantly, these three drugs had no obvious cell toxicity within the concentration ranges that we tested (Fig. 1g) and they did not induce ER stress by themselves (Supplementary Fig. 1f).

### Amoxapine, desloratadine, and maprotiline inhibit all three UPR pathways induced by ER stress

**Fig. 2:**
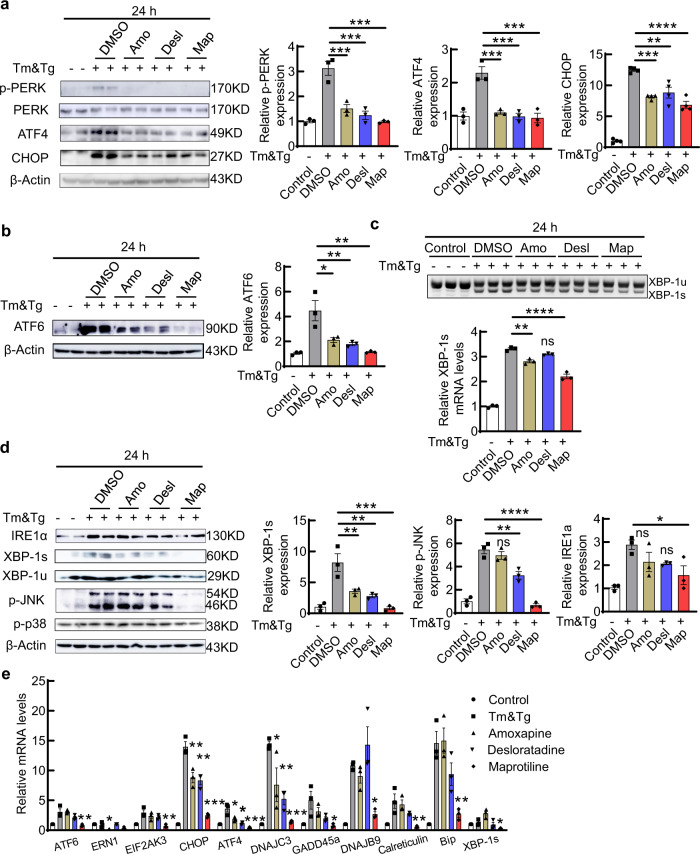
Amo, Desl and Map inhibit the signaling cascades downstream of the three UPR pathways. **a** Immunoblot of HEK293T cells showing the effects of the three compounds on the PERK-CHOP pathway. Quantification of phosphorylated PERK (p-PERK), ATF4 and CHOP 24 h after DMSO, Amo, Desl, or Map (10 µM) treatment in the presence of Tm/Tg (1 µM), relative to control (without Tm/Tg). **b** Immunoblot showing the effects of the three compounds on the ATF6 pathway. Quantification of total ATF6 protein level 24 h after DMSO, Amo, Desl or Map treatment in the presence of Tm/Tg, relative to control (without Tm/Tg). **c** RT-PCR showing the mRNA levels of un-spliced and spliced forms of XBP-1 (XBP-1u and XBP-1s). Quantification of XBP-1s mRNA level 24 h after DMSO, Amo, Desl, or Map treatment in the presence of Tm/Tg, relative to control (without Tm/Tg). **d** Immunoblot showing the effects of the three compounds on the IRE1α-XBP-1/JNK pathway. Quantification of XBP-1s, phosphorylated JNK and IRE1α 24 h after DMSO, Amo, Desl, or Map treatment in the presence of Tm/Tg, relative to control (without Tm/Tg). **e** qPCR mRNA measurement of the UPR genes in HEK293T cells treated for 12 h with DMSO, Amo, Desl, Map (10 µM) in the presence of Tm/Tg (1 µM), relative to control (without Tm/Tg). All data in this figure are presented as means ± s.e.m., *n* = 3 independent replicates, *P < 0.05, **P < 0.01, ***P < 0.001, ****P < 0.0001, one-way ANOVA with Dunnett’s multiple comparisons test. Source data are provided as a Source data file.

To determine whether these compounds inhibit the CHOP branch of UPR preferentially or act as general ER stress modulators that also affect the other two branches, we examined the signaling cascades downstream of the three UPR pathways induced by ER stress after treatment of HEK293T cells with each of these compounds. We again used Tm/Tg (1 µM) to induce ER stress and compared the three hit compounds (10 µM) to DMSO control: (1) The three compounds significantly inhibited PERK phosphorylation and expression of ATF4 and CHOP, indicating upstream modulation of the PERK-CHOP pathway (Fig. 2a). (2) The three compounds also significantly inhibited ATF6 expression (Fig. 2b). (3) Amoxapine and maprotiline likewise significantly inhibited the third UPR pathway downstream of IRE1α activation, as indicated by XBP-1 mRNA splicing (Fig. 2c). All three compounds also downregulated XBP-1s protein level (Fig. 2d). JNK phosphorylation is another downstream effector of IRE1α and contributes to cell death, especially in glaucoma^29,30^. Desloratadine and maprotiline significantly inhibited JNK phosphorylation and maprotiline inhibited IRE1α phosphorylation (Fig. 2d). Lastly, we used qPCR as additional confirmation that these three compounds inhibited various downstream genes of UPR at the mRNA levels; maprotiline showed the most striking modulation of all the UPR pathways (Fig. 2e).

### The three compounds inhibit neuronal ER stress and provide significant neuroprotection in the in vivo mouse traumatic ON crush (ONC) model

**Fig. 3:**
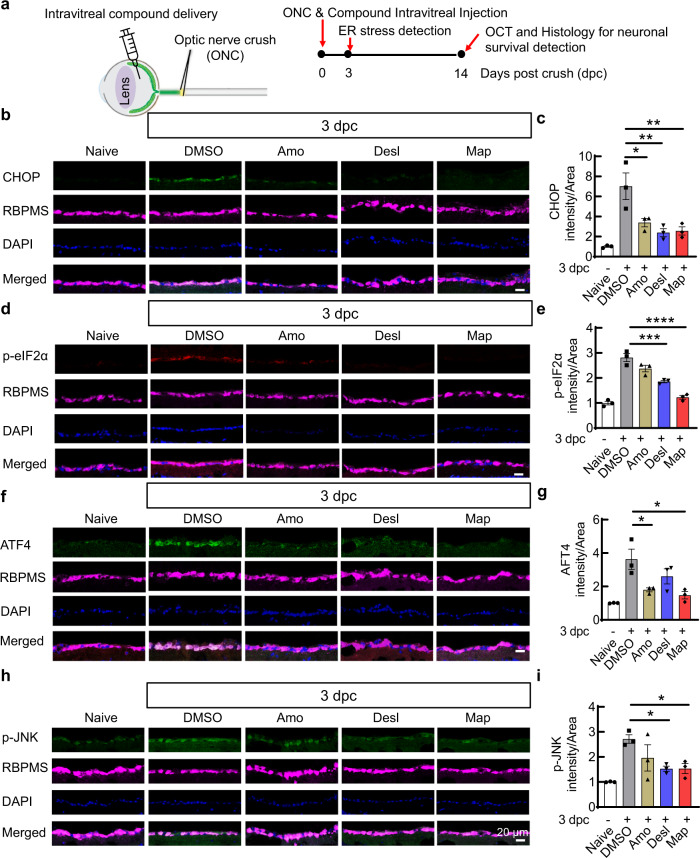
In vivo application of the three hit compounds inhibits ONC-induced ER stress in RGCs. **a** Schematic depicting the mouse ONC model and the timeline of ER stress and neuroprotection evaluation. For compound treatment, each eye received intravitreal injection with 2 µl of 2 mM test compounds once and intraperitoneal (i.p.) injection daily (15 mg/kg). **b**, **d**, **f**, **h** Immunohistochemistry analysis showing the levels of CHOP (**b**), p-eIF2α (**d**), ATF4 (**f**), and p-JNK (**h**) in GCL (ganglion cell layer) of retina sections at 3dpc. **c**, **e**, **g**, **i** Quantification of corresponding fluorescence intensities of CHOP (**b**), p-eIF2α (**d**), ATF4 (**f**), and p-JNK (**h**) in GCL. Data are presented as means ± s.e.m., *n* = 3 mice, *P < 0.05, **P < 0.01, ***P < 0.001, ****P < 0.0001, one-way ANOVA with Dunnett’s multiple comparisons test. Source data are provided as a Source data file.

We previously demonstrated that traumatic ON injury induces ER stress in RGCs at 3 days post crush (3dpc)^14^. To determine the in vivo effects of these three ER stress modulators, we delivered each of the three compounds into mouse eyes by intravitreal injection on the same day after ON crush (ONC) and examined the expression of the key ER stress molecules in RGCs at 3dpc and the survival of RGC somata and axons at 14dpc (Fig. 3a). Consistent with our cell-based in vitro assays, local administration of the three compounds significantly inhibited ONC-induced CHOP and ATF4 expression, and the phosphorylation of eIF2α and JNK in RGCs examined in both retinal sections (Fig. 3b–i) and retinal wholemounts (Supplementary Fig. 2a, b). Interestingly, western Blot assays of ON lysates demonstrated that the three compounds also inhibited ER stress molecules elevated by ONC in the ONs (Supplementary Fig. 2c, d).

**Fig. 4:**
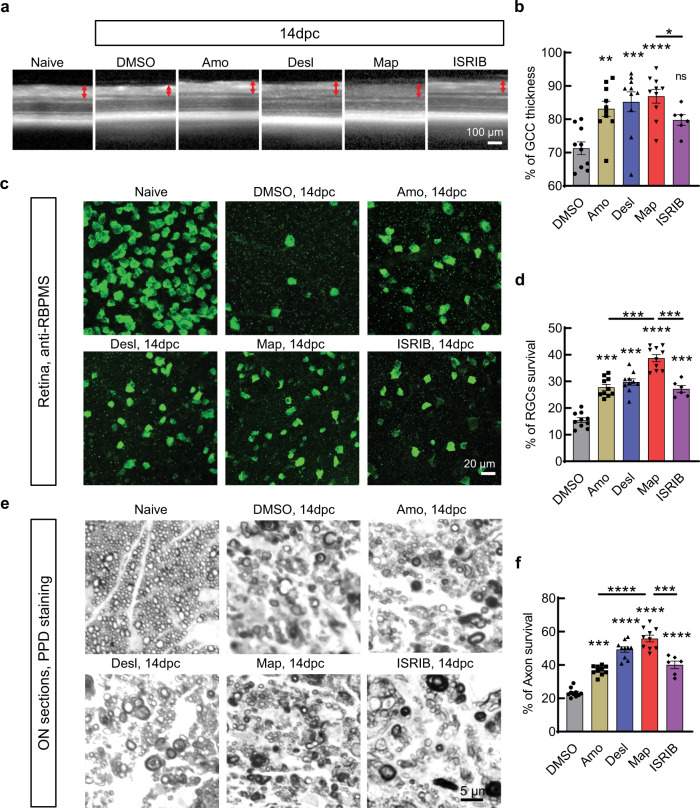
In vivo application of the three hit compounds significantly protects RGC somata and axons after traumatic ONC injury. **a** Representative OCT images of mouse retina in living animals at 14dpc. GCC: ganglion cell complex, including RNFL, GCL, and IPL layers; indicated as double end red arrows. **b** Quantification of GCC thickness measured by OCT at 14dpc, represented as percentage of GCC thickness in the ONC eyes compared to the sham contralateral control eyes. *n* = 10 mice for DMSO, Amo, Desl, and Map; *n* = 6 mice for ISRIB. **c** Representative confocal images of peripheral flat-mounted retinas showing surviving RBPMS + (green) RGCs at 14dpc. **d** Quantification of surviving RGCs at 14dpc, represented as percentage of ONC eyes compared to the sham contralateral control eyes. *n* = 10 mice for DMSO, Amo, Desl, and Map; *n* = 6 mice for ISRIB. **e** Light microscope images of semi-thin transverse sections of ON with PPD staining at 14dpc. **f** Quantification of surviving RGC axons in ON at 14dpc, represented as percentage of crushed ONs compared to the sham contralateral control ONs. *n* = 10 mice for DMSO, Amo, Desl, and Map; *n* = 6 mice for ISRIB. All data in this figure are presented as means ± s.e.m., ****P < 0.0001 ***P < 0.001, **P < 0.01, *P < 0.05, ns: no significance, one-way ANOVA with Dunnett’s multiple comparisons test to compare each treatment to DMSO group. For compound treatment, each eye received intravitreal injection with 2 µl of 2 mM test compounds once and intraperitoneal (i.p.) injection daily (15 mg/kg). Control groups received the same volume of DMSO as vehicle control. Source data are provided as a Source Data file.

ONC is extensively used as a traumatic optic neuropathy model that injures all RGC axons and causes universal RGC and ON degeneration^14,23,31–33^. Therefore, we next used this model to examine the effects of the three ER stress inhibitors on RGC soma and axon survival. We again delivered the three compounds into one of the mouse eyes by intravitreal injection on the same day as ONC, left the contralateral eye as internal control, and then maintained the compound exposure by daily intraperitoneal (i.p.) injection to avoid repeated intravitreal injection. The control group was treated with vehicle (DMSO). We have used optical coherence tomography (OCT) before to image and measure retina thickness in living animals as an accurate in vivo morphological readout for RGC degeneration^34–37^. OCT images at 14dpc showed significant thinning of the ganglion cell complex (GCC), in crushed eyes treated with DMSO compared to contralateral naïve eyes, whereas crushed eyes treated with amoxapine, desloratadine, or maprotiline showed significantly thicker GCC than DMSO-treated eyes (Fig. 4a, b), suggesting significant RGC neuroprotection by these compounds. Histological analysis of post-mortem retina wholemounts and ON semi-thin sections consistently demonstrated significant loss of RGC somata and axons at 14dpc in the DMSO control group, whereas the survival of RGCs and axons was much higher in the compound-treated eyes (Fig. 4c–f). We confirmed the axon protection effect of these compounds by TEM analysis of ON cross sections (Supplementary Fig. 3). ISRIB is a small molecule inhibitor of the PERK pathway identified through cell-based screening^27^. It showed no effect with the reporter cell line (Fig. 1d), but ISRIB provided neuroprotection in the ONC mouse model, to a lesser degree than maprotiline (Fig. 4a–f). Taken together, these results show that in vivo application of each of the three compounds (amoxapine, desloratadine, and maprotiline) effectively inhibits RGC ER stress and significantly protects RGCs and ONs after traumatic ON injury. Because maprotiline shows the most potent and consistent effects on ER stress modulation and neuroprotection both in vitro and in vivo, we focused on characterization of maprotiline in the subsequent experiments.

### Maprotiline significantly promotes both RGC soma and axon survival and preserves visual functions in mouse SOHU glaucoma model

**Fig. 5:**
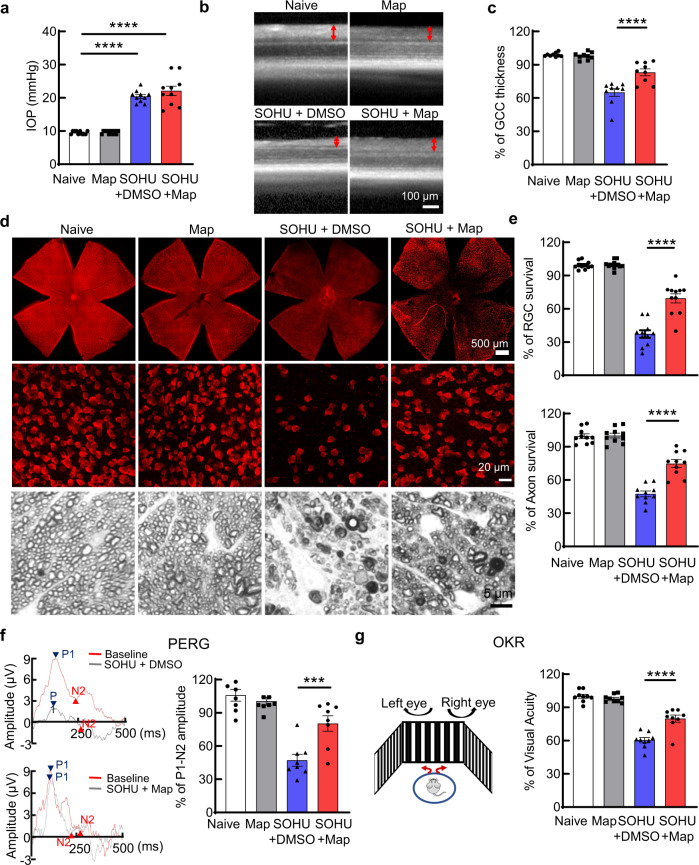
Systemic administration of Map significantly protects RGC somata and axons and preserves visual function in a mouse glaucoma model. **a** IOP measurements at 3wpi. SOHU: intracameral injection of SO to induce ocular hypertension. *n* = 10 mice. **b** Representative OCT images of mouse retina in living animals at 3wpi. GCC is indicated as double end red arrows. **c** Quantification of GCC thickness measured by OCT at 3wpi, represented as percentage of GCC thickness in the SOHU eyes compared to the sham contralateral control eyes. *n* = 9 mice. **d** Upper panel: representative confocal images of wholemounts of the entire retinas. Middle panel: representative confocal images of flat-mounted peripheral retinas showing surviving RBPMS + (red) RGCs at 3wpi. Lower panel: representative light microscope images of semi-thin transverse sections of ON with PPD staining at 3wpi. **e** Quantification of surviving RGC somata in wholemount retinas and axons in ON sections at 3wpi, represented as percentage in SOHU eyes compared to the sham contralateral control eyes. *n* = 10 mice. **f** Left: representative wave forms of PERG at baseline and 3wpi. Right: quantification of P1-N2 amplitude of PERG at 3wpi, represented as percentage in glaucomatous eyes compared to the sham contralateral control eyes. *n* = 8 mice. **g** Left: illustration of OKR measurement of mouse. Right: visual acuity measured by OKR at 3wpi, represented as percentage of cycles/degree value in the glaucomatous eyes compared to the sham contralateral control eyes. *n* = 9 mice. All data in this figure are presented as means ± s.e.m., ***P < 0.001, ****P < 0.0001, one-way ANOVA with Dunnett’s multiple comparisons test. For compound treatment, each eye received retrobulbar injection twice on day 0 and day 10 with 50 µl of 2 mM maprotiline, and daily i.p. injection with maprotiline (15 mg/kg) for 3 weeks after SO injection. Control groups received the same volume of DMSO as vehicle control. Source data are provided as a Source data file.

We previously developed the silicone oil-induced ocular hypertension (SOHU) mouse glaucoma model, which faithfully replicates human secondary glaucoma with persistent elevation of IOP and severe degeneration of RGCs and ON^34,37,38^. To test the effect of maprotiline on glaucomatous neurodegeneration, we generated the SOHU glaucoma model in one eye, used the contralateral eye as sham control, and treated the animals both systemically by i.p. injection + by local retrobulbar injection of compounds or vehicle (DMSO). We performed i.p. injection daily based on the presence of maprotiline in the retina 6 and 24 h after injection (Supplementary Fig. 4a). We did not use intravitreal injection to deliver the drug directly into the eye because intravitreal injection itself can lower IOP and therefore compromise the ocular hypertension glaucoma model. CHOP and ATF4 expression were elevated in glaucomatous RGCs at one week post SO injection (1wpi) in the SOHU model; their expression was significantly inhibited by systematic administration of the three compounds (Supplementary Fig. 4b, c). Next, we focused on maprotiline. Maprotiline treatment did not affect normal IOP in naïve mice, nor elevated IOP in SOHU mice (Fig. 5a). In vivo OCT retinal imaging showed significant thinning of the GCC at 3 weeks post SO injection (3wpi) in the DMSO group, whereas maprotiline treatment significantly increased GCC thickness (Fig. 5b, c). Histological analysis of post-mortem retina wholemounts and semi-thin ON sections consistently demonstrated significantly greater RGC soma and axon survival in the maprotiline group than in the DMSO group (Fig. 5d, e). We confirmed the axon protection effect of maprotiline in the SOHU glaucoma model by TEM analysis of ON cross sections and CTB tracing in wholemount ONs (Supplementary Fig. 5).

The clinical significance of neuroprotection depends on preserving neuronal function. Therefore, we also investigated whether maprotiline preserves visual function in the glaucomatous mice. The optokinetic tracking response (OKR) is a natural reflex that objectively assesses mouse visual acuity^39,40^. Another important electrophysiological assessment of RGC function is the pattern electroretinogram (PERG), in which the ERG responses are stimulated with contrast-reversing horizontal bars alternating at constant mean luminance^41^. We used both techniques, which are well established in our lab^34,35,37,38^, to evaluate maprotiline’s effect on glaucomatous eyes. Consistent with our morphological and histological results, maprotiline significantly preserved visual function in glaucomatous eyes, as demonstrated by improved amplitude of PERG (Fig. 5f) and visual acuity (Fig. 5g) compared to the DMSO control group. Taken together, these results show that maprotiline treatment achieves significant RGC and ON neuroprotection and preserves visual functions in a mouse glaucoma model, confirming its potential as a neuroprotectant.

### Histamine receptor H1(HRH1) is a common antagonist target of amoxapine, desloratadine, and maprotiline for ER stress modulation

**Fig. 6:**
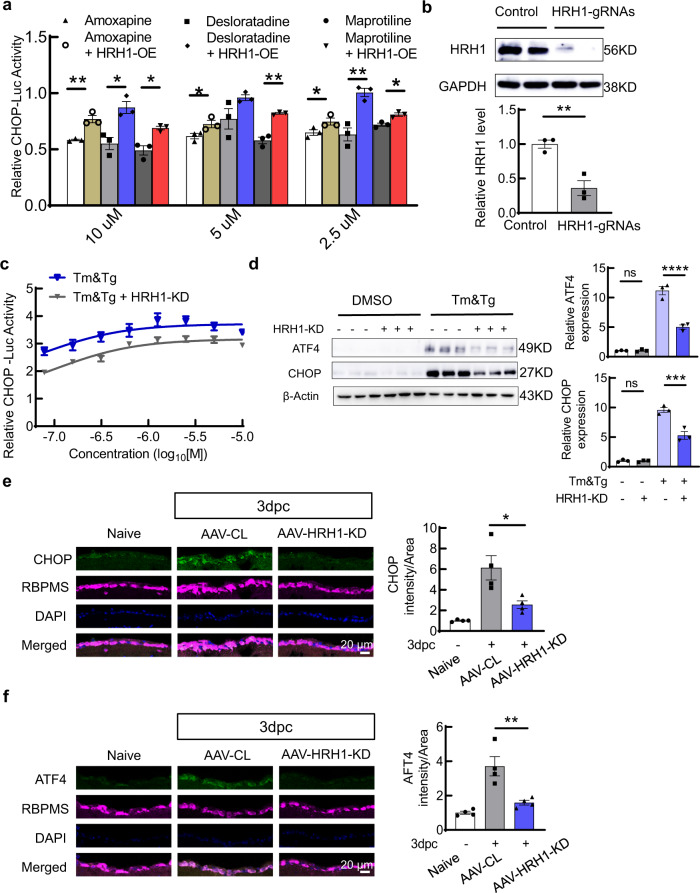
The effects on ER stress modulation of HRH1, the common target of the three hit compounds. **a** Relative CHOP-Luc activities of the three compounds at indicated concentrations in the presence of Tm/Tg (1 µM), with or without HRH1 overexpression (OE), relative to DMSO, 24 h after exposure. *n* = 3 independent replicates. *P < 0.05, **P < 0.01, with a two-tailed unpaired Student’s t-test. **b** Immunoblot of HEK293T cells showing the CRISPR-mediated HRH1 KD and quantification of HRH1 protein levels. *n* = 3 independent replicates. **P < 0.01, with a two-tailed unpaired Student’s t-test. **c** Relative CHOP-Luc (*n* = 5 independent replicates) activities in response to Tm/Tg at indicated concentrations, with or without HRH1-KD, 24 h after exposure. **d** Immunoblot of HEK293T cells showing the protein levels of ATF4 and CHOP with or without HRH1 inhibition. Quantification of relative protein levels. Data are presented as means ± s.e.m., *n* = 3 independent replicates, ****P < 0.0001, ***P < 0.001, ns: no significance, one-way ANOVA with Dunnett’s multiple comparisons test. **e**, **f** Left: immunohistochemistry analysis showing the levels of CHOP (**e**), and ATF4 (**f**) in GCL of retina sections at 3dpc. Right: quantification of corresponding fluorescence intensities/area in GCL. Data are presented as means ± s.e.m., *n* = 4 mice, *P < 0.05, **P < 0.01, one-way ANOVA with Dunnett’s multiple comparisons test. Source data are provided as a Source data file.

We next explored potential downstream effectors of the hit compounds for ER stress modulation and neuroprotection. We reasoned that since the three ER stress modulators, amoxapine, desloratadine, and maprotiline, have similar chemical structures, they may act on a common downstream target to restore ER homeostasis. Because, intriguingly, all three agents are potent antagonists of HRH1 with high binding affinities^42–45^, we hypothesized that HRH1 inhibition may mediate the effects of these compounds on ER stress modulation. To test this hypothesis, we first overexpressed (OE) human HRH1 (Supplementary Fig. 6a) in the CHOP-Luc reporter cell line by transient transfection. The overexpression of HRH1 significantly but not completely reversed the three compounds’ inhibitory effect on Tm/Tg induced CHOP expression (Fig. 6a), indicating that other mechanisms in addition to HRH1 inhibition may also contribute to the compounds’ effects on ER stress. To test whether other UPR pathways are also modulated by HRH1, we generated a stable XBP-1-Luc HEK293T reporter cell line expressing the human XBP-1 fragment-fused luciferase construct (Supplementary Fig. 6b). This construct contains a 26 nt intron sequence that will be removed from the mRNA by IRE1α upon ER stress. Splicing of the 26 nt intron will allow a shift of the open reading frame in the mRNA to express luciferase, which will serve as a reporter for the activation of IRE1α pathway^46^. This cell line shows consistent dose response to Tg and Tm (Supplementary Fig. 6c–e). Maprotiline also shows a dose-dependent inhibition of XBP-1 splicing induced by Tm/Tg. Overexpression of HRH1 reversed the inhibitory effects of maprotiline (Supplementary Fig. 6f) and amoxapine and desloratadine (Supplementary Fig. 6g) on XBP-1 splicing. Therefore, overexpression of HRH1 significantly blocked the activities of the three ER stress modulators, consistent with their antagonistic effect on this receptor.

We next tested whether blocking HRH1 itself has a similar ER stress modulation effect as the three compounds. First, we generated a pair of gRNAs targeting human HRH1 (Supplementary Fig. 6a) and confirmed the HRH1 knockdown (KD) effect of CRISPR in HEK293T cells (Fig. 6b). We then transfected Cas9 and HRH1 gRNAs into the two reporter cell lines and compared Tm/Tg-induced ER stress with or without HRH1 KD. HRH1 inhibition consistently downregulated Tm/Tg-induced CHOP expression (Fig. 6c) and XBP-1 splicing (Supplementary Fig. 6h). To further confirm the HRH1 KD effect on ER stress, we also examined the protein levels of various UPR molecules and again found that HRH1-KD significantly inhibited Tm/Tg-induced ATF4 and CHOP expression (Fig. 6d); JNK phosphorylation and ATF6 expression (Supplementary Fig. 6i); and XBP-1 mRNA splicing (Supplementary Fig. 6j). We previously demonstrated AAV-mSncg promoter mediated Cas9 expression and CRISPR-mediated gene KD in RGCs in vivo^25^. Using the same strategy, we designed gRNAs targeting mouse HRH1 and injected the mixture of AAV-mSncg-Cas9 + AAV-mouse HRH1-gRNAs (Supplementary Fig. 7a) or AAV-control gRNAs intravitreally into mouse eyes (Supplementary Fig. 7b). The endogenous HRH1 mRNA level was detected in some mouse RGCs (Supplementary Fig. 7c). Immunostaining showed HRH1 protein levels to be more extensive in RGCs, and crush injury, but not glaucoma, decreased protein expression in RGCs (Supplementary Fig. 7d, e). AAV-mediated CRISPR KD of HRH1 significantly blocked CHOP and ATF4 expression induced by ONC injury (Fig. 6e, f). Taken together, our studies demonstrated that the three hit compounds inhibit ER stress through their antagonistic effects on their common target, HRH1, suggesting that HRH1 inhibition may provide a potential neuroprotection strategy.

### HRH1 KD provides significant neuroprotection in two mouse optic neuropathy models

**Fig. 7:**
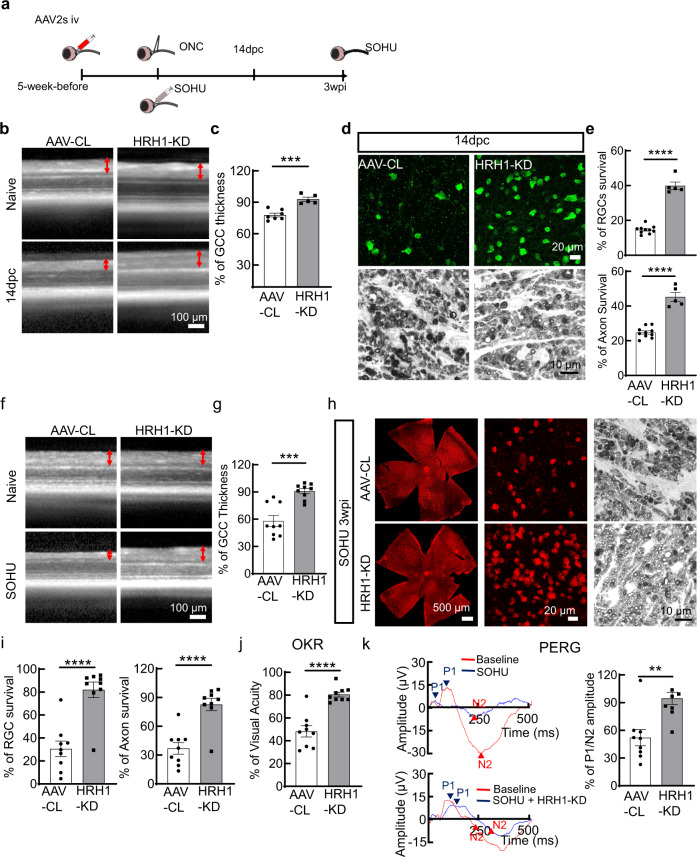
AAV-mediated in vivo CRISPR KD of HRH1 in RGCs significantly protects RGCs and ONs and preserves visual function in two optic neuropathy models. **a** Schematic illustration of the timelines of AAV injection and evaluation of neuroprotection in two optic neuropathy models. **b** Representative OCT images of mouse retina in at 14dpc. GCC is indicated as double end red arrows. **c** Quantification of GCC thickness measured by OCT at 14dpc. AAV-CL, *n* = 7 mice; HRH1-KD, n = 5 mice. **d** Upper panel: representative confocal images of the wholemount retinas; Lower panel: representative light microscope images of semi-thin transverse sections of ON with PPD staining at 14dpc. **e** Quantification of surviving RGC somata in wholemount retinas and axons in ON sections at 14dpc. *n* = 10 mice for AAV-CL, Amo; *n* = 5 mice for HRH1-KD. **f** Representative OCT images of mouse retina in living mice with SOHU glaucoma at 3wpi. GCC is indicated as double end red arrows. **g** Quantification of GCC thickness measured by OCT at 3wpi. *n* = 9 mice. **h** Representative confocal images of the whole retina and enlarged peripheral retina showing surviving RBPMS + (red) RGCs at 3wpi; and representative light microscope images of semi-thin transverse sections of ON with PPD staining. **i** Quantification of surviving RGC somata in wholemount retinas and axons in ON sections at 3wpi, represented as percentage of SOHU eyes compared to the sham contralateral control eyes. *n* = 9. **j** Visual acuity measured by OKR at 3wpi. *n* = 9 mice. **k** Left: representative wave forms of PERG at baseline and 3wpi. Right: quantification of P1-N2 amplitude of PERG at 3wpi. *n* = 9 mice. All data in this figure are presented as means ± s.e.m., **P < 0.01, ***P < 0.001, ****P < 0.0001, with a two-tailed unpaired Student t-test. Source data are provided as a Source data file.

We next investigated whether HRH1 inhibition also furnishes neuroprotection in two mouse optic neuropathy models. We injected the mixture of AAV-mSncg-Cas9 + AAV-mouse HRH1-gRNAs or AAV-control gRNAs intravitreally into one of a mouse’s eyes five weeks before ONC (for traumatic ON injury model) or SO intracameral injection (SOHU glaucoma model) and used the contralateral eye as sham control (Fig. 7a). In the ONC model, in vivo OCT imaging showed that the GCC was significantly thicker in HRH1 KD mice than control mice (Fig. 7b, c). Histological analysis of post-mortem retina wholemounts and ON sections consistently demonstrated significant protection of RGC somata and axons by HRH1 KD (Fig. 7d, e). In the SOHU glaucoma model, HRH1 KD also showed a significantly thicker GCC and greater survival of RGC somata and axons than controls (Fig. 7f–i). Importantly, we also confirmed visual function preservation by HRH1 KD, measured by OKR and PERG (Fig. 7j, k). Therefore, like maprotiline treatment, blocking HRH1 significantly protects RGCs and ONs and preserves visual functions in two mouse optic neuropathy models, indicating the promising therapeutic potential of HRH1 inhibition in traumatic and glaucomatous neurodegeneration. Investigation of the long-term safety of maprotiline and HRH1 KD on naïve mouse retinas revealed no RGC or ON degeneration one month after systemic maprotiline administration or three months after local retina AAV-mediated CRISPR HRH1 KD (Supplementary Fig. 8a, b). We also found no immune cell infiltration in retina and ON after either of these treatments (Supplementary Fig. 8c–f).

### Maprotiline blocks axon injury-induced intracellular Ca^2+^ influx by inhibiting HRH1-mediated ER Ca^2+^ release

**Fig. 8:**
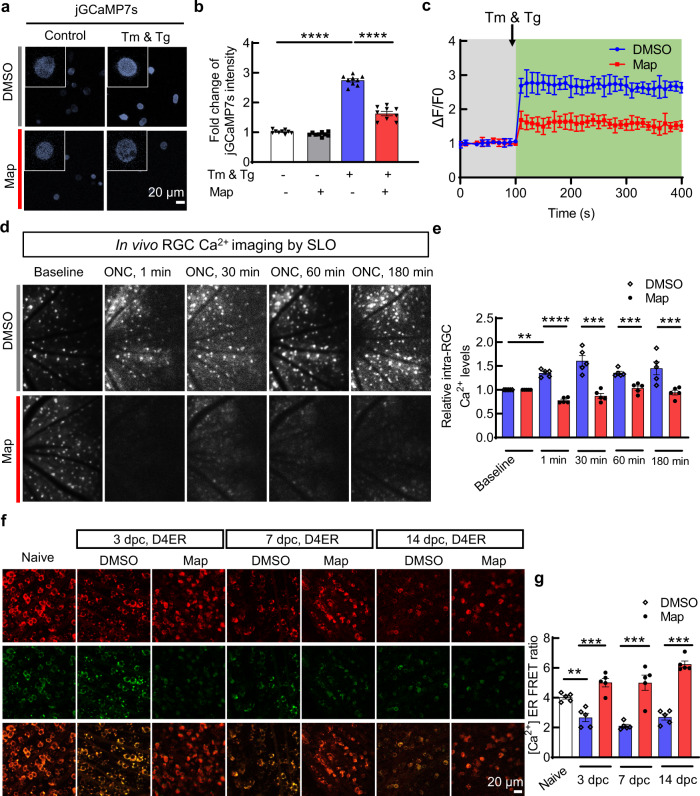
Map effectively inhibits axon injury-induced intracellular Ca^2+^ influx and ER Ca^2+^ release. **a** jGCaMP7s expression in HEK293T cells as an indicator of cytoplasmic Ca^2+^ levels. **b** Quantification of jGCaMP7s fluorescence intensity, 0.5 h after DMSO or Map treatment in the presence of Tm/Tg, represented as fold changes to DMSO-treated control cells. *n* = 9 independent replicates. **c** Cytoplasmic Ca^2+^ influx in HEK293T cells immediately after Tm/Tg (1 µM) treatment, *n* = 3 independent replicates. **d** In vivo retina Ca^2+^ imaging by SLO in living animals expressing jGCaMP7s in RGCs shows cytoplasmic Ca^2+^ influx in RGCs induced by axon injury. **e** Quantification of relative intra-RGC Ca^2+^ levels, represented as fold changes to the baseline fluorescence intensity of DMSO-treated retina. *n* = 5 mice. **f** Ex vivo measurement of ER Ca^2+^ retention in RGCs expressing D4ER after ONC. The D4ER FRET ratio value (Citrine/ECFP) reflects the steady-state ER Ca^2+^ concentration. **g** Quantification of ER Ca^2+^ levels in RGCs expressing D4ER, represented as FRET ratios. *n* = 5 mice. All data in this figure are presented as means ± s.e.m., **P < 0.01, ***P < 0.001, ****P < 0.0001, one-way ANOVA with Dunnett’s multiple comparisons test. Source data are provided as a Source data file.

HRH1 is a Gq protein-coupled receptor that can activate phospholipase C (PLC)-IP_3_ pathway; it leads to ER Ca^2+^ release through IP3 receptors and cytosol Ca^2+^ influx (Supplementary Fig. 9a)^47^. ON injury is well-known to induce rapid intra-axonal Ca^2+^ influx that leads to axon degeneration^48,49^. Ca^2+^ release from the ER, the major intracellular Ca^2+^ storage site, contributes to the deleterious intra-axonal Ca^2+^ influx^50–52^, and at the same time, the disturbance of ER Ca^2+^ homeostasis is also an important initiator of the ER stress^17,18^. We reasoned that maprotiline may block ER Ca^2+^ release by inhibiting HRH1, and therefore restore ER Ca^2+^ homeostasis and prevent ER stress. Using pharmacologic small molecule inhibitors of signaling downstream of HRH1, we confirmed that blocking PLC or IP_3_, but not DAG-PKC, decreased CHOP and XBP-1 activation induced by Tm/Tg (Supplementary Fig. 9b, c), suggesting that HRH1-mediated ER Ca^2+^ release contributes to ER stress. Moreover, PLC inhibitor U-73122 protected RGC somata and axons in vivo after ONC injury (Supplementary Fig. 9d). Therefore, we investigated the effect of maprotiline on the intracellular and ER Ca^2+^ levels. First, we transfected genetically encoded Ca^2+^ sensor jGCaMP7s^53^ into HEK293T cells (Fig. 8a), and confirmed that maprotiline significantly blocked Tm/Tg-induced intracellular Ca^2+^ influx (Fig. 8b, c). Next, we assessed Ca^2+^ influx in RGCs after ONC in vivo: we confirmed efficient AAV-mediated jGCaMP7s expression in RGCs (Supplementary Fig. 10a); and then recorded in vivo RGC Ca^2+^ imaging in living animals by scanning laser ophthalmoscope (SLO) at different time points after ONC injury. Within minutes after ONC, intra-RGC Ca^2+^ levels were significantly elevated, indicating rapid Ca^2+^ influx induced by axon injury, whereas maprotiline significantly decreased intra-RGC Ca^2+^ levels at both early time points (Fig. 8d, e) and later time points (Supplementary Fig. 10b). This significant decrease indicates efficient blocking of Ca^2+^ influx by maprotiline, presumably through inhibition of HRH1-mediated Ca^2+^ release from the ER. To definitively prove this mechanism, we measured intra-ER Ca^2+^ levels by expressing a FRET-based ER Ca^2+^ sensor, D4ER^54,55^, driven by the mSncg promoter^25^, in mouse RGCs specifically (Supplementary Fig. 10c). We confirmed that ONC significantly depleted ER Ca^2+^ of RGCs, whereas, in dramatic contrast, maprotiline maintained ER Ca^2+^ concentration at much higher levels (Fig. 8f, g). Taken together, our data demonstrated that maprotiline blocks ER Ca^2+^ release through HRH1 inhibition, by which it restores ER homeostasis, prevents deleterious intracellular Ca^2+^ influx and ultimately protects injured/diseased RGCs and ONs.

## Discussion

The present experiments first identified three FDA approved medicines, two antidepressants (amoxapine and maprotiline) and one antihistamine/anti-allergy drug (desloratadine), as potent blockers of ER stress-induced CHOP expression, and then as general modulators of all three UPR pathways and as effective neuroprotectants. Although it is formally possible that these compounds inhibit the PERK-CHOP pathway directly and indirectly affect the other two pathways through cross talk, we favor a model in which they have a global effect inhibiting the UPR by modulating upstream signaling of ER stress. Indeed, this notion receives support from our finding that inhibition of HRH1, a common antagonistic target of all three drugs^42–45^, also achieved comparable ER stress inhibition and in vivo neuroprotection in both traumatic ON injury and ocular hypertension glaucoma models. It is known that HRH1 activation leads to Ca^2+^ release from ER^47^ and that depletion of ER Ca^2+^ worsens ER function and induces ER stress^17,18^. Using both cytosol and ER-targeted Ca^2+^ biosensors, we found that maprotiline inhibits ER inducer (Tm/Tg)-induced and axon injury-induced ER Ca^2+^ release and cytosol Ca^2+^ influx both in cultured cells and mouse RGCs in vivo. The restoration of Ca^2+^ homeostasis then attenuates the global UPR signaling. We found maprotiline to be the most potent of the three drugs in modulating ER stress based on in vitro cell-based assays and that its systemic administration caused no detectable toxicity on the normal retina, but significantly protected RGCs and ONs and visual functions in mouse disease models of glaucoma and traumatic injury. The potent in vivo neuroprotection of maprotiline correlates with its potent ER stress modulation, further evidence for its on-target mechanism of action.

Our studies not only identified potent ER stress modulators and effective neuroprotectants, but also revealed a molecular mechanism that regulates ER stress: maprotiline (possibly amoxapine and desloratadine as well) restores ER homeostasis and keeps the three UPR pathways in check by blocking HRH1-mediated Ca^2+^ release from the ER. Many small molecule modulators of ER stress that target the signaling molecules in the three main UPR pathways have been developed for different purposes, some of them are neuroprotective^22^ but none target intracellular Ca^2+^ signaling, which is critical for many neurodegenerative diseases associated with axon degeneration^48–52^. Other HRH1 antagonists may also be able to modulate ER stress and therefore merit further exploration as neuroprotectants. However, some of the other HRH1 antagonists^45^ that we tested inhibited ER stress only at high concentration (Fig. 1d, Supplementary Fig. 1e), and overexpression of HRH1 only partially blocked the three hit compounds’ effect (Fig. 6a). These results suggest that other mechanisms in addition to HRH1 may also be responsible for amoxapine/desloratadine/maprotiline-mediated ER stress inhibition, possibly through other receptors that are also modulated by these compounds. Interestingly, maprotiline has previously been shown to be neuroprotective in Huntington’s disease, potentially through mitochondrial protection and anti-apoptotic mechanisms^56^. Our in vivo findings establish maprotiline as a candidate neuroprotectant and HRH1 as a potential therapeutic target for glaucoma, and possibly for neurodegenerative diseases more generally. Because their safety profiles, pharmacokinetics, and pharmacodynamics, including penetration of blood–brain barrier, are well-known, and because of their extensive clinical usage, maprotiline and/or HRH1 inhibition is a promising pharmacological approach for neuroprotection that can be readily translated to pre-clinical studies in large animals and evaluation in human patients. To this end these compounds may be even more attractive if sustained, local delivery to the eye is pursued, thereby minimizing the potential for systemic side effects.

In this study, we constructed two ER stress reporter HEK293T cell lines that stably express human CHOP promoter-driven luciferase or fused human XBP-1 fragment-luciferase containing a 26 nt intron sequence that will be removed upon ER stress and IREα activation to allow luciferase expression. Through a small scale HTS with the CHOP-Luc reporter line and further validation with both CHOP-Luc and XBP-1-Luc reporter lines, we demonstrated that this powerful strategy efficiently identifies ER stress modulators. We previously found that the two ER stress molecules, CHOP and XBP-1, play opposing roles in glaucomatous degeneration: deletion of CHOP and activation of XBP-1 protect diseased RGCs and ON synergistically^14,23,24^. In this study, the three hit compounds inhibited all three UPR branches, which may not be desirable for neuroprotection because of the inhibition of the IRE1α-XBP1 pathway. A more selective blocker of the PERK-eIF2α-ATF4 pathway, such as ISRIB, may, therefore, be more valuable. Using a murine CHOP promoter-luciferase CHO cell line, another group confirmed that ISRIB inhibits CHOP expression^28^. Unfortunately, ISRIB did not have a significant inhibition effect with our luciferase reporter cell line driven by a human CHOP promoter (Fig. 1d), although it showed significant neuroprotection in the ONC model (Fig. 4), indicating low sensitivity of the reporter line. The much larger murine CHOP promoter (8.5 kb) in the CHO cell line may be more sensitive to compounds or have more cis-regulatory components than the human CHOP promoter (~1 kb) in our HEK cell line, but they do share similar “hits”, including GSK2606414 and trifluoperazine^28^. Cross-checking hit compounds with these two reporter lines will be worthwhile to further confirm CHOP inhibitory effects. Small molecular modulators that inhibit CHOP but activate XBP-1 may furnish even better neuroprotection than those acting by a single mechanism. Encouraged by recent success in identifying preferential activators of IRE1α/XBP-1s and ATF6 with a counter screening strategy^57,58^, we are currently pursuing complementary cell-based HTS using these two reporter lines with much larger chemical libraries to identify advanced ER stress modulators that inhibit the CHOP pathway but activate the XBP-1 pathway.

In summary, we identified three FDA-approved drugs as potent ER stress modulators and effective neuroprotectants through a small scale of HTS, a strategy that warrants further application in identifying additional ER stress modulators. We found that both systemic administration of maprotiline and locally applied CRISPR-mediated RGC-specific HRH1 inhibition achieve significant neuroprotection and visual function recovery in in vivo mouse models of glaucoma and traumatic ON injury. Based on the demonstration of their molecular target and mechanism that we provide in this report and their well-established safety, pharmacological and clinical usage profiles, maprotiline, its structural analogs, and HRH1 antagonists appear to be highly promising candidates for thorough pre-clinical and clinical evaluation as neuroprotectants.

## Methods

### Animals

C57BL/6J WT (#000664) mice (7–9 weeks old, male) were purchased from Jackson Laboratories (Bar Harbor, Maine) and housed in standard cages on a 12-h light–dark cycle with room temperature at 25 ± 2 °C and humidity between 40 and 60%. All experimental procedures were performed in compliance with animal protocol (#32093) approved by the IACUC at Stanford University School of Medicine.

### Constructs

The phCHOP-Luciferase (−954) construct containing human CHOP promoter driven-luciferase was originally made by Dr. Pierre Fafournoux^59^ and given by Dr. Shigeru Takahashi^60^. The XBP1-Luciferase construct was from Dr. Albert Koong^46^, containing the luciferase gene fused downstream of an XBP1 fragment containing the 26 nt intron, splicing of the intron by IRE1 under ER stress results in a frameshift and luciferase translation. The coding regions of D4ER^54,55^ (a gift from Dr. Paola Pizzo, Department of Biomedical Sciences, University of Padua, Italy) and jGCaMP7s (Addgene, #104487) were cloned into our pAM-AAV-mSncg-WPRE backbone containing the RGC-specific mSncg promoter^25^. The human HRH1 coding sequence was cloned from HEK293T cell genomic DNA and inserted into a backbone containing the CMV promoter to create a CMV-HRH1 vector. The AAV2-mSncg-Cas9 and the AAV-U6-sgRNAs-Syn-EGFP have been described before^25^. The mouse HRH1 gRNA sequences are: gRNA1 (5′-GCTCCACAACCCTTCCGAGTA-3′) and gRNA2 (5′-GTCCGTCTTCTCCACAACCCT-3′). The human HRH1 gRNA sequences are: gRNA1 (5′-GTCTCCGTCCTCCTTAACCCC-3′) and gRNA2 (5′-GATTCTCCGTCCTCCTTAAC-3′).

### AAV production and intravitreal injection

AAV2 vector was co-transfected with the pHelper plasmid (Stratagene) and pAAV2 (pACG2)-RC triple mutant into HEK293T cells for 72 h before purification with polyethylene glycol and cesium chloride density gradient centrifugation. The AAV titers were determined by real-time PCR and diluted to 1.5 × 10^12^ vector genome (vg)/ml for mouse intravitreal injection. For intravitreal injection, mice were anesthetized by xylazine and ketamine based on their body weight (0.01 mg xylazine/g + 0.08 mg ketamine/g). A pulled and polished microcapillary needle was inserted into the peripheral retina just behind the ora serrata. Approximately 2 µl of the vitreous was removed to allow injection of 2 µl AAV into the vitreous chamber to achieve 3 × 10^9^ vg/retina.

### ER stress reporter cell lines

HEK293T cells were transiently co-transfected with phCHOP-Luciferase or XBP-1-Luciferase with pEGFP-puro using Lipofectamine 2000 (Invitrogen, Carlsbad, CA) at ratio 5:1 to ensure EGFP positive cells are also luciferase construct positive and puromycin resistant. After a serial selection with puromycin and EGFP expression, multiple stably expressing clones (CHOP-Luc/puro or XBP-1-Luc/puro) were isolated by a serial dilution. After expansion of individual clones, one CHOP-luciferase stable line and one XBP-1-luciferase line were selected based on their responses to Tm/Tg treatment, and maintained by puromycin as stable reporter cell lines used in this study.

### Cell culture and viability assays

HEK293T cells were grown in Dulbecco’s Modified Eagle’s Medium (DMEM) (Invitrogen, 11995081) supplemented with 100 μg/mL streptomycin and 100 units/mL penicillin (Gibco, 15140122), and 10% fetal bovine serum (FBS) (Invitrogen, 10437-028). Cells were maintained under standard tissue culture conditions (5% CO_2_, 37 °C). PolyJet™ (SignaGen Laboratories, SL100688) transfection reagent was used for transient cell transfection. For detection of relative cellular viability levels, cells (10000/well) were seeded into poly-d-lysine coated 96-well plates (Falcon, 353072) and treated as described. Then, MTT (5 mg/mL, 10 μL/well) (MedChemExpress, HY-15924) was added. After incubation at 37 °C for 3–4 h, DMSO was added to dissolve the precipitate, and the absorbances were determined at 570 nm by a Tecan Infinite M1000 Pro plate reader.

### Cell-based HTS

The HTS to identify CHOP expression inhibitors was performed at the Stanford High-Throughput Bioscience Center with small molecule libraries containing 4846 total known bioactive, FDA approved drugs and clinical trial compounds; libraries included Biomol FDA, Biomol ICCB, Microsource (MS) Spectrum, Sigma LOPAC, and NIH Clinical Collection (NIH-CC). All the compounds were run in 7-point dose response based on a previous publication^26^; except the NIH Clinical collection (446 compounds) which were tested in duplicate due to the small quantity of compounds on hand. Most of the NIH-CC was screened at 10 µM, but this varied per compound. The Z’ of the assay was 0.5, details on instrumentation can be found here: https://med.stanford.edu/htbc/equipment/liquid.html. We used the Pin Tool to transfer 100 nL of the compounds into 50 µL final volume. Therefore, we did a 500-fold dilution of the stock plates, which were at 10, 5, 2.5, 1.25, 0.625, 0.3125, and 0.156 mM resulting in final concentrations of 20, 10, 5, 2.5, 1.25, 0.625, and 0.3125 µM (for most of the compounds but not all). The final DMSO concentration was 0.2% for all wells. Briefly, CHOP-Luc cells were seeded in 384-well plates (Greiner Bio-One CELLSTAR™) at a density of 2 × 10^5^ cells per well in 40 µL medium and cultured for 24 h before treatment with tunicamycin (Tm) + thapsigargin (Tg) at 1 µM in 10 µL medium followed by adding 100 nL of one of the testing compounds using a Staccato SciClone ALH3000 small molecule liquid handling system (Caliper Life Sciences) and V&P Scientific 384 pin tools. AeraSeal sterile adhesive microplate seals (Excel Scientific; Victorville, CA) were used to seal plates that were incubated at 5% CO2, 37 °C for 24 h, when luciferase activity was assayed by adding 10 μL of Bright^TM^-Glo Luciferase reagent (Promega) to each well and detected by a Tecan Infinite M1000 Pro plate reader. Percentage of CHOP-Luc inhibition = 100%−(Compound value−DMSO control value)/(Tm/Tg value−DMSO control value) × 100%. The compounds achieving >30% of CHOP-Luc inhibition were considered as “hits”. Luciferase activity of all the tested compounds was determined by meta-analysis of luciferase activity in different cell lines under the control of a generic or other promoters. Any compound that appeared in more than 3 non-related luciferase screens was likely a toxic compound or luciferase inhibitor and therefore eliminated. There was no true cutoff, other than an IC_50_ < 20 µM (or the highest concentration tested). Most of these compounds were toxic, but we did not specifically examine toxicity because our goal here was to eliminate the non-specific hits regardless of whether they were luciferase inhibitors or toxic compounds.

### Commercial compounds

Thapsigargin (Sigma, T9033), Tunicamycin (Sigma, T7765), Maprotiline (Sigma, M9651), Amoxapine (MedChemExpress, HY-B0991), Desloratadine (MedChemExpress, HY-B0539), Desipramine (Sigma, D3900), Trifluoperazine (Sigma, T8516), Clomipramine (Sigma, C7291), Amitriptyline (Sigma, A8404), Quetiapine (Sigma, Q3638), Olanzapine (MedChemExpress, HY-14541), Doxepin (MedChemExpress, B078), Loxapine (Sigma, L106), Norquetiapine (Sigma, 07849), dimethyl sulfoxide (DMSO) (Sigma, D8418), 2-APB (MedChemExpress, HYW009724), U-73122 (MedChemExpress, HY13419), Go 6983 (MedChemExpress, HY13689), GSK2606414 (Sigma, 516535), ISRIB (MedChemExpress, HY-12495).

### Protein preparation and immunoblotting

Cell and tissue lysates were prepared in RIPA buffer (Themo Fisher Scientific, 89901) and supplemented with Halt Protease inhibitor cocktail (Themo Fisher Scientific, PI78437). The total protein concentration of lysates was measured by the Pierce BCA Protein Assay Kit (Thermo Fisher Scientific, 23227). 20 µg protein of each sample were denatured at 95 °C for 15 min in 100 mM DTT + 1× Laemmli buffer before being separated by SDS-PAGE. We then transferred the protein samples to 0.2 µm nitrocellulose membranes (Bio-Rad, 1610097) and blocked with 5% BSA for 2 h. Subsequently, the membranes were incubated with primary antibodies (1:1000) overnight at 4 °C. After washing in TBST, these membranes were incubated with horseradish peroxidase-conjugated secondary antibodies (Cell Signaling, 7074S and 7076S, 1:1000) and visualized using a GE-AI600 imaging system. Images were quantified with ImageJ software. The primary antibodies used were PERK (Cell Signaling, 3192S), p-PERK (Cell Signaling, 3179 S), eIF2a (Cell Signaling, 5324S), p-eIF2a (Cell Signaling, 3597L), ATF4 (Cell Signaling, 11815S), CHOP (Cell Signaling, 2895S), ATF6 (Cell Signaling, 65880T), IRE1a (Cell Signaling, 3294T), IRE1 (phosphor-S724) (Thermo Fisher, PA116927), phospho-SAPK/JNK (Thr183/Tyr185) (Cell Signaling, 9251S), phospho-p38 MAPK (Thr180/Tyr182) (Cell Signaling, 9215S), β-Actin (sigma, A5441), XBP-1s (Biolegend, 647502), XBP-1 (Santa Cruz, sc-8501), anti-RBPMS (Custom made at ProSci Inc).

### RT-PCR for XBP1 splicing assay and Q-PCR for ER stress genes

Cells were plated into poly-d-lysine coated 6-well plates (Fisher, 353046) and treated as indicated at 37 °C with 5% CO_2_. The total RNA was isolated using Trizol (Thermo Fisher Scientific, 10296010) and 500 ng RNA was reverse transcribed using the High-Capacity cDNA Reverse Transcription Kit (Thermo Fisher Scientific, 4374966) to acquire total cDNA. The XBP-1 mRNA splicing primers (forward primer 5′-GGGGCTTGGTATATATGTGG-3′, reverse primer 5′-CCTTGTAGTTGAGAACCAGG-3′) were utilized to amplify the XBP-1 amplicon containing the 26 nt intron that will be released by IRE1α upon ER stress, with Q5 High-Fidelity DNA Polymerase (NCB, M0491L). PCR products (2 µg) were resolved on 2.5% agarose gels, visualized with GelRed (Biotium), and quantified by ImageJ (NIH). The relative mRNA expression levels of target genes were detected by PowerUP SYBR Green Master Mix (Thermo Fisher, A25776) and C1000 TOUCH CYCLER w/48 W FS RM PCR System (Bio-rad, 1851148). Thermal cycles were 95 °C for an initial 5 min followed by 40 cycles of denaturation at 95 °C for 15 s, annealing at 60 °C for 30 s and extension at 72 °C for 60 s. Transcripts were normalized to GAPDH and all measurements were performed in triplicate. Primers used were: GAPDH, forward 5′-GTCTCCTCTGACTTCAACAGCG-3′ and reverse 5′-ACCACCCTGTTGCTGTAGCCAA-3′; ATF6, forward 5′ TGGAAGCAGCAAATGAGACG-3′ and reverse 5′-TGAGGAGGCTGGAGAAAGTG-3′; ERN1, forward 5′-CGAACGTGATCCGCTACTTC-3′ and reverse 5′-ATGTTGAGGGAGTGGAGGTG-3′; EIF2AK3, forward 5′-GTCCCAAGGCTTTGGAATCTGTC-3′ and reverse 5′-CCTACCAAGACAGGAGTTCTGG-3′; CHOP, forward 5′-ACCAAGGGAGAACCAGGAAACG-3′ and reverse 5′-TCACCATTCGGTCAATCAGAGC-3′; ATF4, forward 5′-GTCCCTCCAACAACAGCAAG-3′ and reverse 5′-TGTCATCCAACGTGGTCAGA-3′; DNAJC3, forward 5′-GCCTGCATCTGCTTTATGCT-3′ and reverse 5′-TCTGCAAGGCTGTGAAGAGA-3′; GADD45a, forward 5′-GGAGGAAGTGCTCAGCAAAG-3′ and reverse 5′-ACATCTCTGTCGTCGTCCTC-3′; DNAJB9, forward 5′-GGAAGGAGGAGCGCTAGGTC-3′ and reverse 5′-ATCCTGCACCCTCCGACTAC-3′; Calreticulin, forward 5′-CGATGATCCCACAGACTCCA-3′ and reverse 5′-CCGTCCATCTCTTCATCCCA-3′; Bip, forward 5′-GCCTGTATTTCTAGACCTGCC-3′ and reverse 5′-TTCATCTTGCCAGCCAGTTG-3′; XBP-1s, forward 5′-CTCCAGAGACGGAGTCCAAG-3′ and reverse 5′-CACCTGCTGCGGACTC-3′.

### ON crush model

The ON was exposed intraorbitally while care was taken not to damage the underlying ophthalmic artery, and crushed with a jeweler’s forceps (Dumont #5; Fine Science Tools, Foster City, California) for 5 s approximately 0.5 mm behind the eyeball. Eye ointment containing neomycin (Akorn, Somerset, New Jersey) was applied to protect the cornea after surgery. For compound treatment, each eye received intravitreal injection with 2 µl of 2 mM test compounds once and intraperitoneal (i.p.) injection daily (15 mg/kg) for 14 days after ONC. Control groups received the same volume of DMSO as vehicle control.

### SOHU glaucoma model and IOP measurement

Mice were anesthetized by an intraperitoneal injection of Avertin (0.3 mg/g) and received the SO (Alcon Laboratories, 1000 mPa.s) injection at 9–10 weeks of age. Prior to injection, one drop of 0.5% proparacaine hydrochloride (Akorn, Somerset, New Jersey) was applied to the cornea to reduce its sensitivity during the procedure. A 32 G needle was tunneled through the layers of the cornea at the superotemporal side close to the limbus to reach the anterior chamber without injuring lens or iris. Following this entry, ~2 µl silicone oil (1000 mPa.s, Silikon, Alcon Laboratories, Fort Worth, Texas) was injected slowly into the anterior chamber using a homemade sterile glass micropipette, until the oil droplet expanded to cover most areas of the iris (diameter ~1.8–2.2 mm). After the injection, veterinary antibiotic ointment (BNP ophthalmic ointment, Vetropolycin, Dechra, Overland Park, Kansas) was applied to the surface of the injected eye. The contralateral control eyes received mock injection with 2 µl normal saline to the anterior chamber. Throughout the procedure, artificial tears (Systane Ultra Lubricant Eye Drops, Alcon Laboratories, Fort Worth, Texas) were applied to keep the cornea moist. For compound treatment, each eye received retrobulbar injection twice on day 0 and day 10 with 50 µl of 2 mM maprotiline, and daily *i.p*. injection with maprotiline (15 mg/kg) for 3 weeks after SO injection. Control groups received the same volume of DMSO as vehicle control.

The IOP of both eyes was measured by the TonoLab tonometer (Colonial Medical Supply, Espoo, Finland) according to product instructions under a sustained flow of isoflurane (3% isoflurane at 2 L/min mixed with oxygen) delivered to the nose by a special rodent nose cone (Xenotec, Inc., Rolla, Missouri). 1% Tropicamide Sterile Ophthalmic Solution (Akorn, Somerset, New Jersey) was applied three times at 3-min intervals to fully dilate the pupils (about 10 min) before taking measurements. During this procedure, artificial tears were applied to keep the cornea moist. Since IOP measurement requires pupil dilation, which essentially relieves the ocular hypertension during the period of pupil dilation, we only measure IOP 3 weeks after SO injection immediately before sacrificing the animals in the ND (no dilation) SOHU model that we described before^37^.

### LC-MS analysis of maprotiline in retina

Retinal tissues were homogenized with 100 µL of pre-chilled 20% acetonitrile and then diluted 2-fold with blank mouse plasma. An aliquot of 20 µL of diluted retina homogenate was extracted with 100 µL of methanol:acetonitrile (5:95, v-v) containing the internal standard (Verapamil). The mixture was shaken on a shaker for 15 min and then centrifuged at 3220 × *g* for 15 min. An aliquot of 70 µL of the supernatant was mixed with 70 µL of water for the injection to the LC-MS. Calibration standards and quality control samples were prepared by spiking 2 µL of the test compound into 18 µL of blank mouse plasma, and the resulting plasma was processed with the unknown samples in the same batch. The extracts were analyzed by a Shimadzu LC-30AD interfaced to a Sciex API 5000 system. The extracts were injected onto an ACE 3 C18 column (50 × 2.1 mm, 3.0 µm) and separated by the gradient elution using water with 10 mM ammonium acetate (A) and acetonitrile with 0.1% formic acid (B) as mobile phases. The gradient program started at 10% B, held for 0.2 min, ramped to 95% B at 1.5 min, remained at 95% at 2.4 min, dropped to 10% B at 2.45 min, and stayed at 10% B till 3.2 min. The mass spectrometer was operated in positive electrospray ionization under the multiple reaction monitoring (MRM) mode for the detection of the maprotiline (278.277-to-250.2 *m*/*z*) and the internal standard (455.346-to-165 *m*/*z*). The calibration curve fitted by linear regression was used to quantify the analytes in the matrix using Analyst software 1.6.2 (Sciex).

### Immunohistochemistry of whole mount and cross sections of retina

After perfusion fixation with 4% PFA in PBS, mice eyeballs and ONs were dissected out and post-fixed with 4% PFA for 2 h at room temperature. Retinas were dissected out for whole-mount retina immunostaining. For cryo-section with Leica cryostat, the eyeballs and ONs were embedded in tissue-tek OCT (Sakura) on dry ice for subsequent cryo-section. The sections were blocked with 10% goat serum (Sigma, G9023) for 2 h before incubating with primary antibodies: RBPMS 1:4000, others 1:200 overnight at 4 °C. After washing 3 times with PBS, samples were incubated with secondary antibodies (1:400; Jackson ImmunoResearch, West Grove, Pennsylvania) at room temperature for 2 h. Tissues were washed with PBS 3 times before mounting with Fluoromount-G (SouthernBiotech, Alabama). Confocal images were obtained by a Zeiss LSM 800 microscope (Carl Zeiss Microscopy).

### Intracellular Ca^2+^ imaging in HEK293T cells

HEK293T cells were seeded at 10% confluence the day before transfection in poly-D-lysin-coated glass bottom 35 mm dishes (MatTek, P35GC1.510C). Two hours prior to transfection, media was removed and 1 mL of FBS-free DMEM media was added to each well. We then mixed 2 µg of the jGCaMP7s plasmid in 100 μl DMEM. In a separate tube, we diluted 4 μl PolyJet™ (SignaGen Laboratories, SL100688) reagent in 100 μl with DMEM, and incubate for 10 min. The complete mixture of DNA and PolyJet was incubated for another 15 min before being added to the HEK293T cells. Six hours later, the media was changed to 1 ml/well culture media. The next day, cytosolic Ca^2+^ was imaged in cells transiently transfected with jGCaMP7s through a Zeiss LSM 800 confocal microscope. Dynamic intracellular Ca^2+^ influx in response to Tm/Tg-induced ER stress was measured every 10 s for 100 s before Tm/Tg treatment to acquire baseline fluorescence (*F0*) and for 400 s after Tm/Tg administration to acquire *F1*. Δ*F* = *F1−F0*. Data were analyzed with ImageJ.

### In vivo RGC Ca^2+^ imaging with SLO

The mice were intravitreally injected with AAV2-mSncg-jGCaMP7s (9 × 10^9^ vg/retina) 4 weeks before imaging. The mice were anesthetized by xylazine and ketamine after dark adaptation for 30 min. Mydriasis was achieved by applying a drop of 1% tropicamide solution and a drop of 2.5% phenylephrine hydrochloride solution, which prevents pupillary contraction during recording. Right after ON crush, the mice were placed on a 3D-printed mouse holder with a 37 °C heater, and a custom-made +10D mouse contact lens (3.0 mm diameter, 1.6 mm BC, PMMA clear, Advanced Vision Technologies) attached to keep the cornea from drying. The retinal fundus was imaged by the Heidelberg Spectralis SLO/OCT system (Heidelberg Engineering, Germany) with a 55° lens using the fluorescein angiography scanning mode under the same sensitivity (sensitivity 75–85) and high-resolution (1536 × 1536 pixels).

### Ex vivo ER Ca^2+^ imaging in retina explants

ER calcium levels were measured using the Förster resonance energy transfer (FRET)-based ER targeted calcium sensor, D4ER^54,55^. Mouse received intravitreal injection of AAV2-mSncg-D4ER to express D4ER in RGCs in vivo 4 weeks before ON crush injury, as well as intravitreal injection of DMSO (vehicle) or maprotiline compound. For imaging, retinas were dissected out at 3, 7 or 14dpc and plated onto laminin (Sigma, L2020) and poly-D-lysin-coated glass bottom dishes (MatTek, P35GC1.510C) and maintained in Neurobasal-A medium (ThermoFisher Scientific, 10888022) supplemented with L-glutamine (Gibco, 25030-081), penicillin/streptomycin (Gibco, 15140122) and B-27 (ThermoFisher Scientific, 0080085SA). Retina explants were imaged using a Zeiss LSM 800 microscope. ER[Ca^2+^] levels were determined by exciting D4ER at 440 nm to record the emitted light at 465–485 nm and 530–550 nm, and analyzed with ImageJ.

### RGC counting

Whole-mount retinas were immunostained with the RBPMS antibody, 6–8 fields randomly sampled from peripheral regions of each retina using a 40X lens with a Zeiss M2 epifluorescence microscope, and RBPMS + RGCs counted by Volocity software (Quorum Technologies). The percentage of RGC survival was calculated as the ratio of surviving RGC numbers in injured eyes compared to contralateral uninjured eyes. The investigators who counted the cells were masked to the treatment of the samples.

### ON semi-thin sections and quantification of surviving axons

ONs were post-fixed in situ with 2% glutaraldehyde and 2% PFA in 0.1 M PBS. Semi-thin (1 µm) cross sections of the ON 2 mm distal to the eye (globe) were collected. The sections were stained with 1% PPD for 0.5 h before washing with methanol: isopropanol (1:1) 3 times × 10 min and then mounted with Fluoromount-G. Four sections of each ON were imaged through a 100× lens of a Zeiss M2 epifluorescence microscope to cover the entire area of the ON without overlap. Two areas of 21.4 µm × 29.1 µm were cropped from the center of each image, and the surviving axons within the designated areas counted manually using ImageJ. After counting all the images taken from a single nerve, the mean of the surviving axon number was calculated for each ON. The mean of the surviving axon number in the injured ON was compared to that in the contralateral control ON to yield a percentage of axon survival value.

### Transmission electron microscope (TEM) imaging and quantification of surviving axons in ON ultrathin cross sections

70 nm ultrathin sections were collected onto formvar-coated copper grids and dried overnight. Sections were then stained with uranyl acetate for 30 min, washed in PBS, and then stained with lead citrate for 7 min. Sections were again washed and dried before observing under TEM. The cross-sections of the entire ON were examined and imaged randomly without overlap at 4000× with 11.6 μm × 11.6 μm frames on a JEOL JEM-1400 TEM microscope (JEOL USA, Inc., Peabody, MA). For each ON, 25–45 images were taken to cover the whole area of the ON. Axons were counted manually with ImageJ’s Cell Counter plugin.

### CTB tracing in wholemount ON and imaging

Intravitreal injection of CTB was performed 48 h before perfusion of the animals with 4% PFA in PBS. The ONs were carefully dissected with fine forceps and scissors and cleared with a modified iDISCO method^61^: wash with PBS for 4 × 30 min; then immersed in a series of 20%, 40%, 60%, 80%, and 100% methanol in PBS for 30 min at each concentration; dichloromethane (DCM)/methanol (2:1) for 30 min; 100% DCM for 30 min and dibenzyl ether (DBE) for 10 min before mounting on slides. Tiled images of the wholemount ON were captured and stitched by a Zeiss LSM 880 confocal laser scanning microscope with 40x/1.0 Oil DIC (Carl Zeiss Microscopy, Thornwood, NY, USA). Positive CTB areas were identified based on a fluorescence intensity greater than the baseline intensity threshold. The percentage of the CTB positive area in the optic nerve was measured by NIH ImageJ.

### Fluorescent in situ hybridization (ISH) of retina cross sections

Fluorescent in situ hybridization (FISH) was performed by using the RNAscope Multiplex Fluorescent Detection Reagents V2 (Advanced Cell Diagnostics, ACD, Hayward, CA, USA) according to the manufacturer’s instructions. RNAscope probe Mm-Hrh1 (491141) was purchased from ACD. Adult mice were perfused with ice-cold 4% PFA/PBS, and eyes were dissected out and fixed in 4% PFA/PBS at 4 °C overnight. The eyes were dehydrated with increasing concentrations of sucrose solution (10%, 20 and 30%) overnight before embedding in OCT on dry ice. Serial cross sections (12 µm) were cut with a Leica cryostat and collected on Superfrost Plus Slides. The sections were pretreated with protease and then subjected to in situ hybridization with RNAscope Multiplex Fluorescent Detection Reagents V2 according to the manufacturer’s instruction (Advanced Cell Diagnostics, Hayward, CA). Briefly, sections were hybridized with the probe solution, followed by amplification and probe detection using TSA plus fluorophores (AKOYA, Marlborough, MA, USA). The sections were mounted with Fluoromount-G (SouthernBiotech, Birmingham, AL, USA). Images were captured by a Zeiss LSM 880 confocal laser scanning microscope with 40×/1.0 Oil DIC (Carl Zeiss Microscopy, Thornwood, NY, USA).

### Spectral-domain optical coherence tomography (SD-OCT) imaging

The mouse retina was scanned by the Heidelberg Spectralis SLO/OCT system (Heidelberg Engineering, Germany) with the ring scan mode centered by the ON head under high-resolution mode (each B-scan consisted of 1536A scans). The ganglion cell complex (GCC) includes retinal nerve fiber layer (RNFL), ganglion cell layer (GCL) and inner plexiform layer (IPL). The average thickness of GCC around the ON head was measured manually with the aid of Heidelberg software.

### Pattern electroretinogram (PERG) recording

After anesthetization and pupil dilation, PERG of both eyes was recorded simultaneously with the Miami PERG system (Intelligent Hearing Systems, Miami, Florida) according to manufacturer’s instructions. Two consecutive recordings of 200 traces were averaged to achieve one readout; each trace recorded up to 1020 ms. The first positive peak in the waveform was designated as P1 and the second negative peak as N2. The amplitude was measured from P1 to N2.

### Optokinetic tracking response (OKR)

Mice were placed on a platform in the center of four 17-inch LCD computer monitors (Dell, Phoenix, AZ), with a video camera above the platform to capture the movement of the mouse. A rotating cylinder with vertical sine wave grating was computed and projected to the four monitors by OptoMotry software (Cere- bralMechanics Inc, Lethbridge, Alberta, Canada). The sine wave grating, settled at 100% contrast and speed of 12 degrees per second, provides a virtual-reality environment to measure the spatial acuity (cycle/degree) of the left eye when rotated clockwise and the right eye when rotated counterclockwise. The maximum frequency (cycle/degree) that the mouse could track was identified and recorded by investigators masked to treatment. The relative percentages of visual acuity were calculated as the ratio of maximum frequency in disease eye compared to contralateral control eye.

### Statistical analysis

GraphPad Prism 7 was used to generate graphs and for statistical analyses. Data are presented as means ± s.e.m. Student’s t-test was used for two groups comparison and One-way ANOVA with post hoc test was used for multiple comparisons.

### Reporting summary

Further information on research design is available in the Nature Portfolio Reporting Summary linked to this article.

## Supplementary information

Supplementary Information

Reporting Summary

## Data Availability

The main data supporting the findings of this study are available within the article and its Supplementary Figures. The source data underlying Figs. 1–8 and Supplementary Figs. 1–9 are provided as a Source data file. Specific data *P* values are also included within the Source data file. Additional details on datasets and protocols that support the findings of this study will be made available by the corresponding author. Source data are provided with this paper.

## Source data

Source Data

## References

[1] Tham YC (2014). Global prevalence of glaucoma and projections of glaucoma burden through 2040: a systematic review and meta-analysis. Ophthalmology.

[2] Varma R, Lee PP, Goldberg I, Kotak S (2011). An assessment of the health and economic burdens of glaucoma. Am. J. Ophthalmol..

[3] Nickells RW, Howell GR, Soto I, John SW (2012). Under pressure: cellular and molecular responses during glaucoma, a common neurodegeneration with axonopathy. Annu. Rev. Neurosci..

[4] Jonas JB (2017). Glaucoma. Lancet.

[5] Weinreb RN (2016). Primary open-angle glaucoma. Nat. Rev. Dis. Prim..

[6] Calkins, D. J. Adaptive responses to neurodegenerative stress in glaucoma. *Prog. Retin. Eye Res*. **84**, 100953 (2021). 10.1016/j.preteyeres.2021.100953 PMC8384979 33640464

[7] Beykin G, Norcia AM, Srinivasan VJ, Dubra A, Goldberg JL (2021). Discovery and clinical translation of novel glaucoma biomarkers. Prog. Retin Eye Res..

[8] Iyer J, Vianna JR, Chauhan BC, Quigley HA (2020). Toward a new definition of glaucomatous optic neuropathy for clinical research. Curr. Opin. Ophthalmol..

[9] Stowell C, Burgoyne CF, Tamm ER, Ethier CR, Lasker IIoA, Glaucomatous Neurodegeneration P. (2017). Biomechanical aspects of axonal damage in glaucoma: a brief review. Exp. Eye Res..

[10] Heijl A (2002). Reduction of intraocular pressure and glaucoma progression: results from the Early Manifest Glaucoma Trial. Arch. Ophthalmol..

[11] Garway-Heath DF (2015). Latanoprost for open-angle glaucoma (UKGTS): a randomised, multicentre, placebo-controlled trial. Lancet.

[12] Anderson DR, Normal Tension Glaucoma S. (2003). Collaborative normal tension glaucoma study. Curr. Opin. Ophthalmol..

[13] Wormald R, Virgili G, Azuara-Blanco A (2020). Systematic reviews and randomised controlled trials on open angle glaucoma. Eye.

[14] Hu Y (2012). Differential effects of unfolded protein response pathways on axon injury-induced death of retinal ganglion cells. Neuron.

[15] Li, S., Yang, L., Selzer, M. E. & Hu, Y. Neuronal endoplasmic reticulum stress in axon injury and neurodegeneration. *Ann. Neurol.* **74**, 768–777 (2013). 10.1002/ana.24005 PMC3963272 23955583

[16] Hu Y (2016). Axon injury induced endoplasmic reticulum stress and neurodegeneration. Neural Regen. Res..

[17] Walter P, Ron D (2011). The unfolded protein response: from stress pathway to homeostatic regulation. Science.

[18] Hetz C, Zhang K, Kaufman RJ (2020). Mechanisms, regulation and functions of the unfolded protein response. Nat. Rev. Mol. Cell Biol..

[19] Han D (2009). IRE1alpha kinase activation modes control alternate endoribonuclease outputs to determine divergent cell fates. Cell.

[20] Costa-Mattioli, M. & Walter, P. The integrated stress response: From mechanism to disease. *Science* **368**, eaat5314 (2020). 10.1126/science.aat5314 PMC8997189 32327570

[21] Han J (2013). ER-stress-induced transcriptional regulation increases protein synthesis leading to cell death. Nat. Cell Biol..

[22] Hetz C, Axten JM, Patterson JB (2019). Pharmacological targeting of the unfolded protein response for disease intervention. Nat. Chem. Biol..

[23] Yang L (2016). Rescue of glaucomatous neurodegeneration by differentially modulating neuronal endoplasmic reticulum stress molecules. J. Neurosci..

[24] Huang H (2017). Neuroprotection by eIF2alpha-CHOP inhibition and XBP-1 activation in EAE/optic neuritiss. Cell Death Dis..

[25] Wang Q (2020). Mouse gamma-Synuclein promoter-mediated gene expression and editing in mammalian retinal ganglion cells. J. Neurosci..

[26] Inglese J (2006). Quantitative high-throughput screening: a titration-based approach that efficiently identifies biological activities in large chemical libraries. Proc. Natl Acad. Sci. USA.

[27] Sidrauski C (2013). Pharmacological brake-release of mRNA translation enhances cognitive memory. eLife.

[28] Halliday M (2017). Repurposed drugs targeting eIF2α-P-mediated translational repression prevent neurodegeneration in mice. Brain.

[29] Urano F (2000). Coupling of stress in the ER to activation of JNK protein kinases by transmembrane protein kinase IRE1. Science.

[30] Fernandes KA (2012). JNK2 and JNK3 are major regulators of axonal injury-induced retinal ganglion cell death. Neurobiol. Dis..

[31] Huang H (2019). AKT-dependent and -independent pathways mediate PTEN deletion-induced CNS axon regeneration. Cell Death Dis..

[32] Yang L (2014). The mTORC1 effectors S6K1 and 4E-BP play different roles in CNS axon regeneration. Nat. Commun..

[33] Miao L (2016). mTORC1 is necessary but mTORC2 and GSK3beta are inhibitory for AKT3-induced axon regeneration in the central nervous system. eLife.

[34] Zhang, J. et al. Silicone oil-induced ocular hypertension and glaucomatous neurodegeneration in mouse. *eLife* **8**, e45881 (2019). 10.7554/eLife.45881 PMC6533060 31090540

[35] Li L (2020). Longitudinal morphological and functional assessment of RGC neurodegeneration after optic nerve crush in mouse. Front. Cell. Neurosci..

[36] Zhang Y (2020). In vivo evaluation of retinal ganglion cells and optic nerve’s integrity in large animals by multi-modality analysis. Exp. Eye Res..

[37] Fang F (2021). Chronic mild and acute severe glaucomatous neurodegeneration derived from silicone oil-induced ocular hypertension. Sci. Rep..

[38] Zhang, J. et al. A reversible silicon oil-induced ocular hypertension model in mice. *J. Vis.Exp.: JoVE* **153**, e60409 (2019). 10.3791/60409 PMC6938455 31789319

[39] Prusky GT, Alam NM, Beekman S, Douglas RM (2004). Rapid quantification of adult and developing mouse spatial vision using a virtual optomotor system. Invest. Ophthalmol. Vis. Sci..

[40] Douglas RM (2005). Independent visual threshold measurements in the two eyes of freely moving rats and mice using a virtual-reality optokinetic system. Vis. Neurosci..

[41] Porciatti V (2015). Electrophysiological assessment of retinal ganglion cell function. Exp. Eye Res..

[42] Canonica GW, Blaiss M (2011). Antihistaminic, anti-inflammatory, and antiallergic properties of the nonsedating second-generation antihistamine desloratadine: a review of the evidence. World Allergy Organ J..

[43] Kanba S, Richelson E (1984). Histamine H1 receptors in human brain labelled with [3H]doxepin. Brain Res.

[44] von Coburg Y, Kottke T, Weizel L, Ligneau X, Stark H (2009). Potential utility of histamine H3 receptor antagonist pharmacophore in antipsychotics. Bioorg. Med. Chem. Lett..

[45] Appl H (2012). Interactions of recombinant human histamine H(1)R, H(2)R, H(3)R, and H(4)R receptors with 34 antidepressants and antipsychotics. Naunyn Schmiedebergs Arch. Pharm..

[46] Papandreou I (2011). Identification of an Ire1alpha endonuclease specific inhibitor with cytotoxic activity against human multiple myeloma. Blood.

[47] Panula P (2015). International union of basic and clinical pharmacology. XCVIII. Histamine receptors. Pharm. Rev..

[48] Knoferle J (2010). Mechanisms of acute axonal degeneration in the optic nerve in vivo. Proc. Natl Acad. Sci. USA.

[49] Ribas VT, Koch JC, Michel U, Bahr M, Lingor P (2017). Attenuation of axonal degeneration by calcium channel inhibitors improves retinal ganglion cell survival and regeneration after optic nerve crush. Mol. Neurobiol..

[50] Stirling DP, Cummins K, Wayne Chen SR, Stys P (2014). Axoplasmic reticulum Ca(2+) release causes secondary degeneration of spinal axons. Ann. Neurol..

[51] Villegas R (2014). Calcium release from intra-axonal endoplasmic reticulum leads to axon degeneration through mitochondrial dysfunction. J. Neurosci..

[52] Orem BC, Rajaee A, Stirling DP (2020). IP3R-mediated intra-axonal Ca(2+) release contributes to secondary axonal degeneration following contusive spinal cord injury. Neurobiol. Dis..

[53] Dana H (2019). High-performance calcium sensors for imaging activity in neuronal populations and microcompartments. Nat. Methods.

[54] Greotti, E., Wong, A., Pozzan, T., Pendin, D. & Pizzo, P. Characterization of the ER-targeted low affinity Ca(2+) probe D4ER. *Sensors* **16**, 1419 (2016). 10.3390/s16091419 PMC5038697 27598166

[55] Kipanyula MJ (2012). Ca2+ dysregulation in neurons from transgenic mice expressing mutant presenilin 2. Aging Cell.

[56] Lauterbach EC (2013). Neuroprotective effects of psychotropic drugs in Huntington’s disease. Int. J. Mol. Sci..

[57] Grandjean JMD (2020). Pharmacologic IRE1/XBP1s activation confers targeted ER proteostasis reprogramming. Nat. Chem. Biol..

[58] Plate, L. et al. Small molecule proteostasis regulators that reprogram the ER to reduce extracellular protein aggregation. *eLife* **5**, e15550 (2016). 10.7554/eLife.15550 PMC4954754 27435961

[59] Bruhat A (2000). Amino acids control mammalian gene transcription: activating transcription factor 2 is essential for the amino acid responsiveness of the CHOP promoter. Mol. Cell Biol..

[60] Yamazaki T (2010). Regulation of the human CHOP gene promoter by the stress response transcription factor ATF5 via the AARE1 site in human hepatoma HepG2 cells. Life Sci..

[61] Renier N (2014). iDISCO: a simple, rapid method to immunolabel large tissue samples for volume imaging. Cell.

