# Supplementary material for: Maprotiline restores ER homeostasis and rescues neurodegeneration via Histamine Receptor H1 inhibition in retinal ganglion cells
Source: Nat Commun. 2022 Nov 10;13:6796. doi: 10.1038/s41467-022-34682-y (PMC9649812; doi:10.1038/s41467-022-34682-y)
Supplement: Supplementary file 1 — Supplementary Information [file 41467_2022_34682_MOESM1_ESM.pdf]

Supplementary Information for

Maprotiline restores ER homeostasis and rescues neurodegeneration via Histamine Receptor H1 inhibition in retinal ganglion cells

Wei Chen[1,2,§], Pingting Liu[1,§], Dong Liu[1], Haoliang Huang[1], Xue Feng[1], Fang Fang[1,3], Liang Li[1], Jian Wu[1,4], Liang Liu[1], David E. Solow-Cordero[5], and Yang Hu[1*]

Corresponding author: Yang Hu
Email: huyang@stanford.edu

This PDF file includes:

Supplementary Figures 1-10

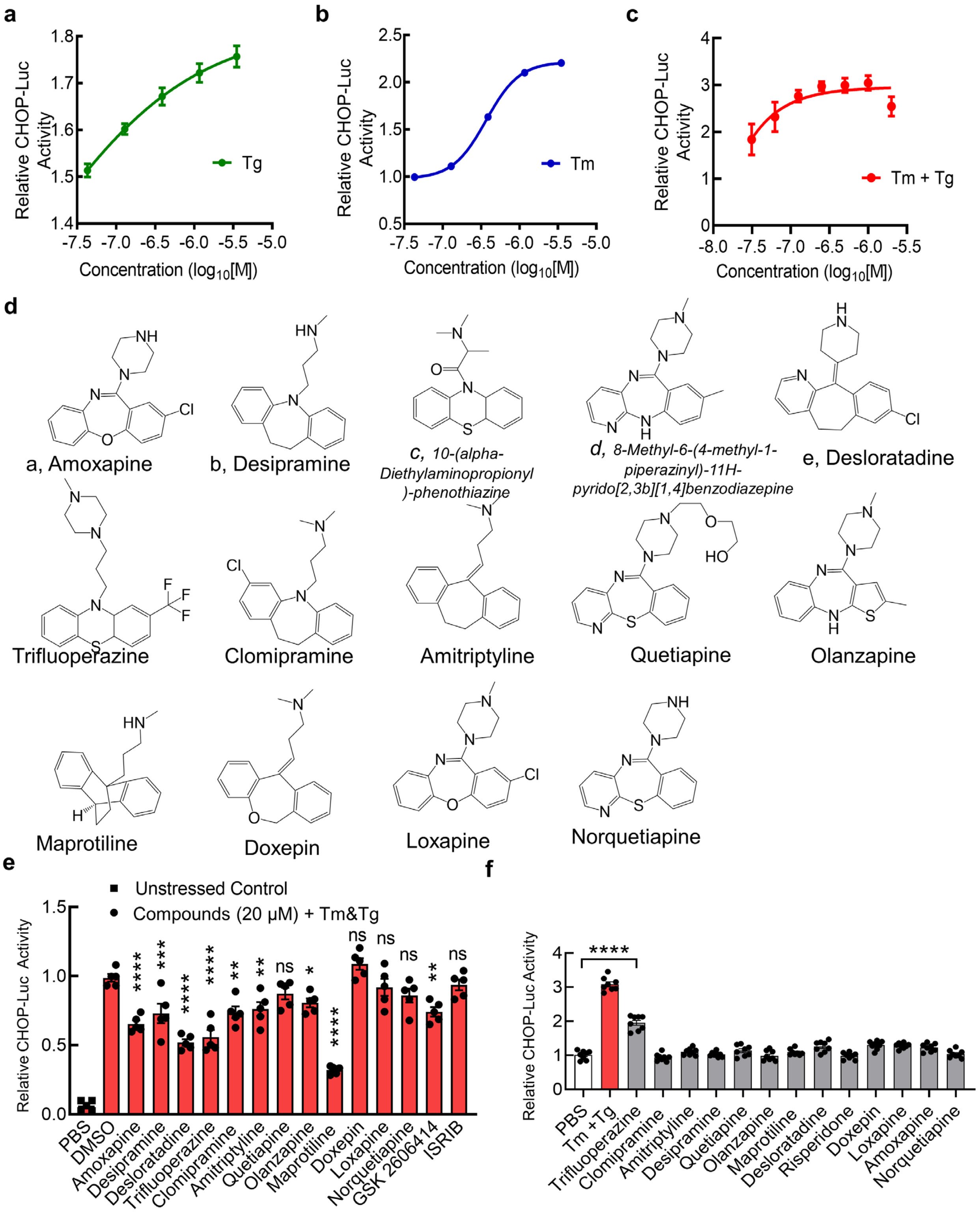

Supplementary Figure 1. CHOP-Luc cell-based assay. Relative CHOP-Luc activities at indicated concentrations of thapsigargin (Tg) (**a**) tunicamycin (**b**), and Tm/Tg (**c**) relative to

baseline, 24 hours after exposure. *n* = 4 independent replicates. **d** Chemical structures of hit compounds a-e and 9 additional analog compounds with similar structures. **e** Relative CHOP-Luc activities of individual tested compounds at 20 μM in the presence of Tm/Tg (1 μM) relative to DMSO, 24 hours after exposure. *n* = 3 independent replicates. **f** Relative CHOP-Luc activities of individual tested compounds at 10 μM, compared to Tm/Tg at 1 μM, relative to baseline, 24 hours after exposure. *n* = 8 independent replicates. All data in this figure are presented as means ± s.e.m, *P < 0.05, **P < 0.01, ***P < 0.001, ****P < 0.0001, ns: no significance, one-way ANOVA with Dunnett's multiple comparisons test. Source data are provided as a Source Data file.

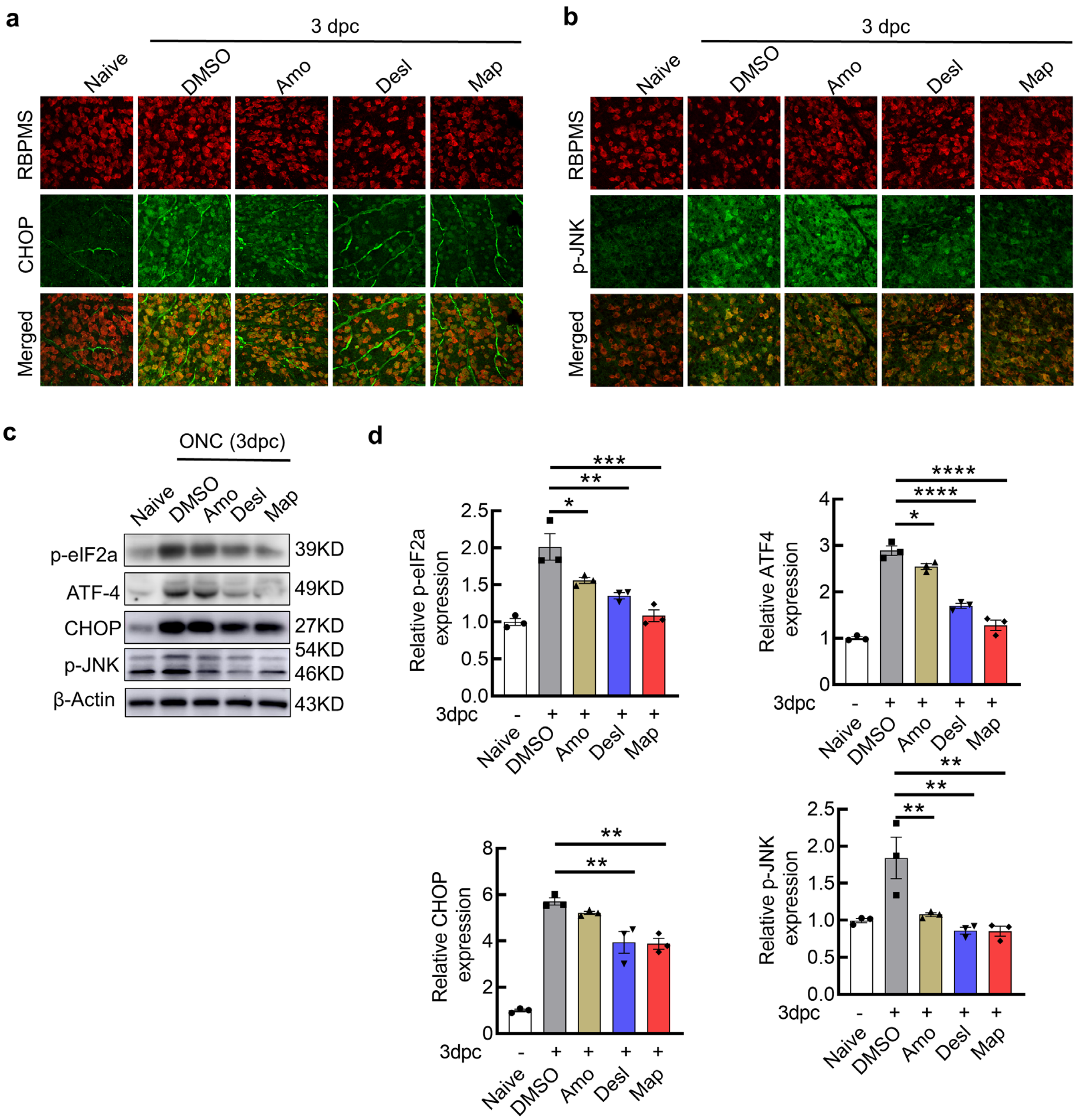

Supplementary Figure 2. ER stress inhibition in RGCs and ONs by the three compounds. (a, b) Representative confocal images of flat-mount retinas showing CHOP or p-JNK signals in RGCs at 3dpc. **c** Immunoblot of mouse ONs showing protein levels of ER stress molecules in ONs at 3dpc. **d** Quantification of relative protein levels of ER stress molecules in ONs at 3dpc. Data are presented as means ± s.e.m, $n = 3$ independent replicates, ****$P < 0.0001$, ***$P < 0.001$, **$P <$

0.01, *$P < 0.05$, one-way ANOVA with Dunnett's multiple comparisons test. Source data are provided as a Source Data file.

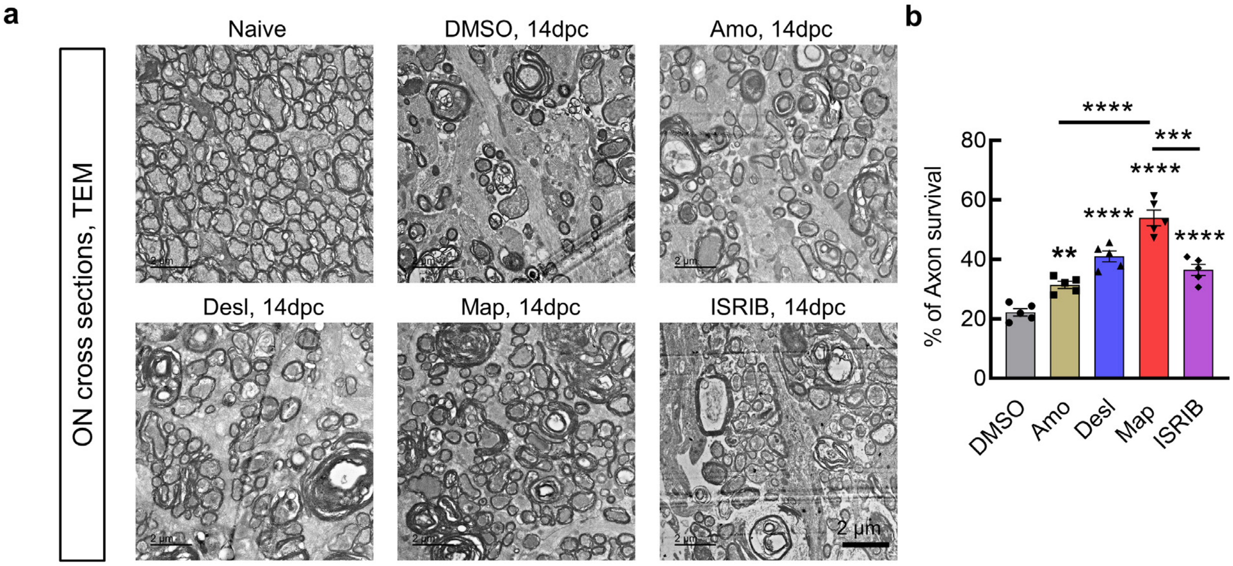

Supplementary Figure 3. Significant RGC axon protection by the three hit compounds in ONC revealed by TEM. a Representative TEM images of transverse sections of ON (4,000x magnification). **b** Quantification of surviving RGC axons in ONs at 14dpc, represented as percentage of crushed ONs compared to the sham contralateral control ONs. Data are presented as means ± s.e.m, n = 5 mice, ****$P < 0.0001$, ***$P < 0.001$, **$P < 0.01$, one-way ANOVA with Dunnett's multiple comparisons test. Source data are provided as a Source Data file.

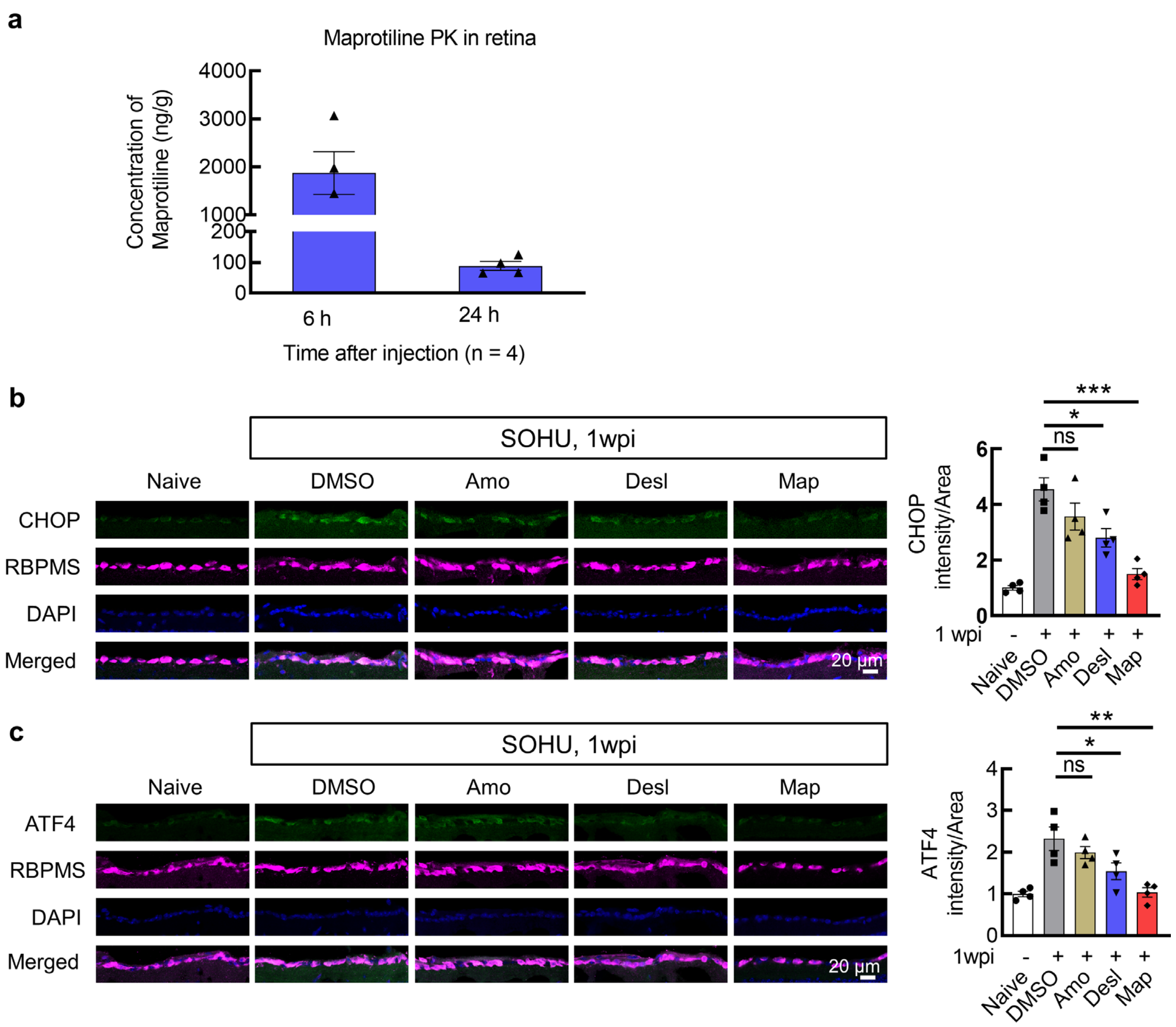

Supplementary Figure 4. *In vivo* application of three hit compounds inhibits SOHU-induced ER stress in RGCs. a LC-MS analysis of retina exposure of Map after *i.p.* injection at 6 and 24 hours. (**b, c**) Immunohistochemistry results showing the levels of CHOP (**b**) and ATF4 (**c**) in GCL of retina sections at 1wpi in SOHU mouse eyes. Quantification of corresponding fluorescence intensity/area in GCL. Data are presented as means ± s.e.m, n = 4 independent replicates, *P < 0.05, **P < 0.01, ***P < 0.001, ns: no significance, one-way ANOVA with Dunnett's multiple comparisons test. Source data are provided as a Source Data file.

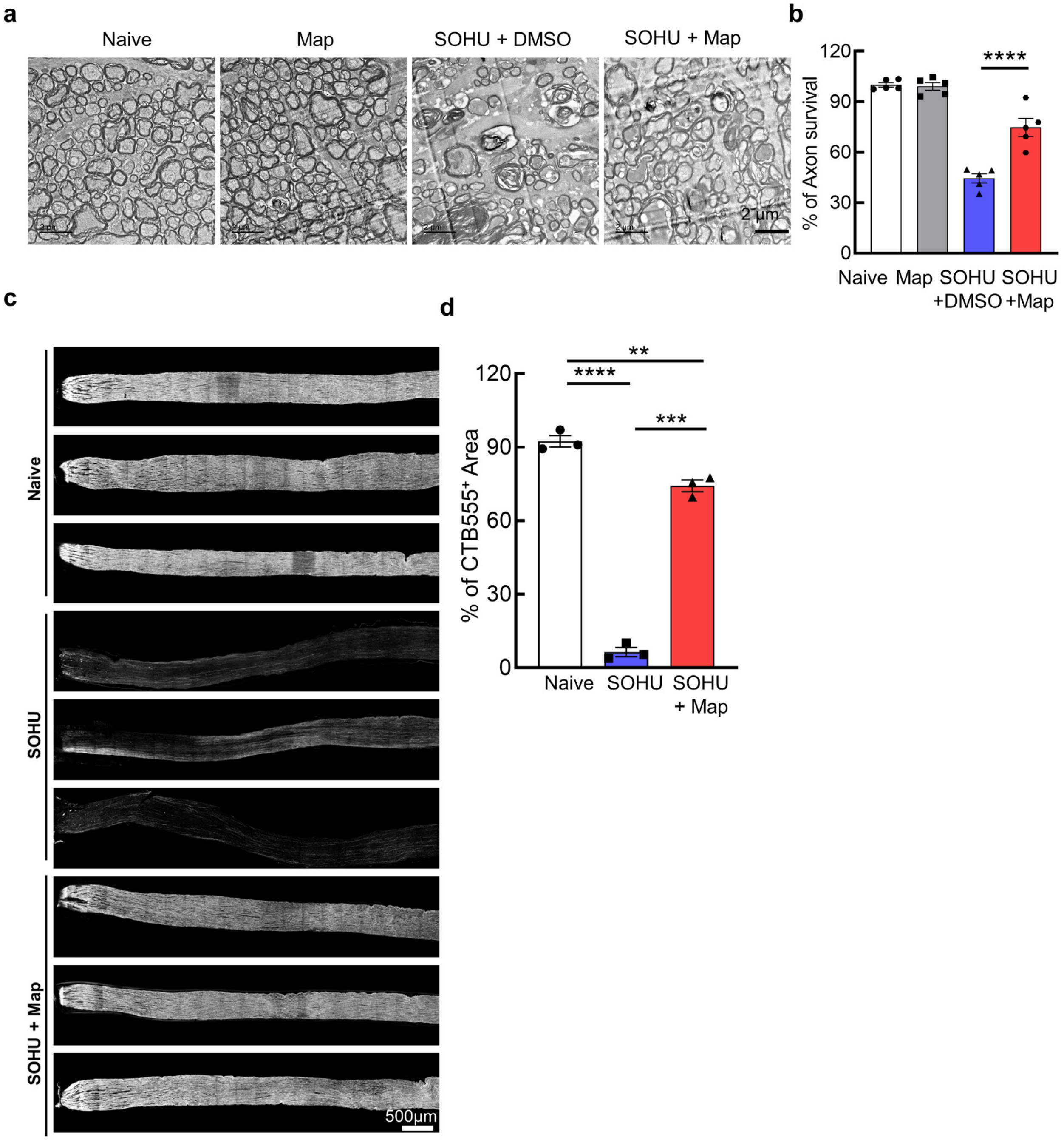

Supplementary Figure 5. Significant RGC axon protection by systemic administration of Map in SOHU glaucoma model. a Representative TEM images of transverse sections of ON (4,000x magnification). **b** Quantification of surviving RGC axons in ONs at 3wpi, represented as percentage of SOHU ONs compared to the sham contralateral control ONs. Data are presented as

means ± s.e.m, $n = 5$ mice, ****$P < 0.0001$, one-way ANOVA with Dunnett's multiple comparisons test. **c** Confocal images of wholemount ONs with anterograde CTB tracing at 3wpi. Scalebar **d** Quantification of the percentage of CTB-labeled area to total ON area. $n = 3$ mice. Data are presented as means ± s.e.m, ****P < 0.0001, ***P < 0.001, **P < 0.01, one-way ANOVA with Dunnett's multiple comparisons test. Source data are provided as a Source Data file.

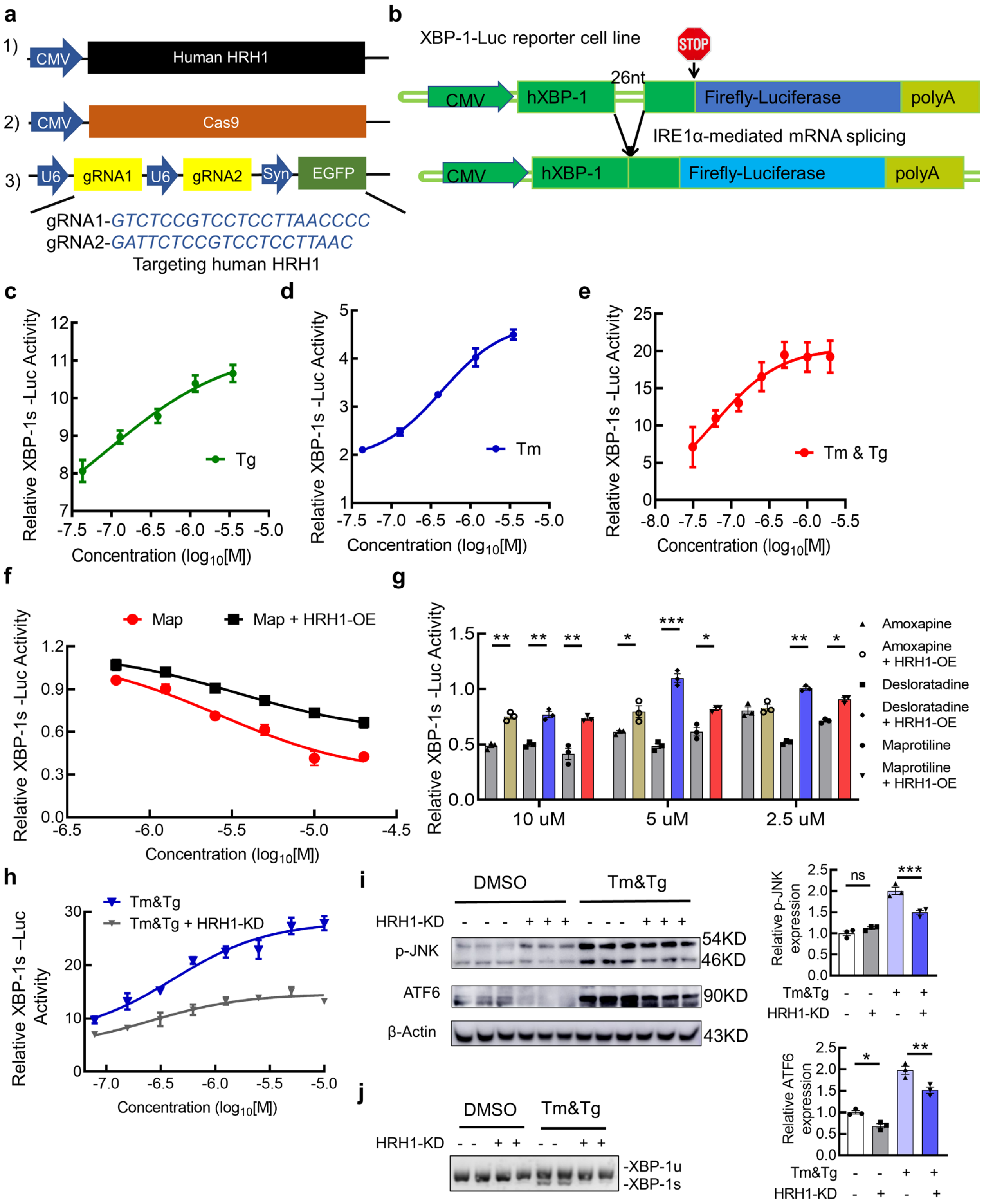

Supplementary Figure 6. The effect of HRH1 on XBP-1 splicing. a Schematic depicting the vectors for HRH1 OE and CRISPR-mediated KD. **b** Schematic illustrating the XBP-1-Luc stable

cell line to report IRE1α activity and XBP-1 splicing. (**c-e**) Relative XBP-1s-Luc activities at indicated concentrations of Tg, Tm, or Tm/Tg in relative to baseline, 24 hours after exposure. Data are presented as means ± s.e.m, $n = 4$ independent replicates. **f** Relative XBP-1s-Luc activity of Map at indicated concentrations in the presence of Tm/Tg (1 μM), with or without HRH1-OE, relative to DMSO, 24 hours after exposure. $n = 3$ independent replicates. **g** Relative XBP-1s-Luc activities at indicated concentrations of Amo, Desl and Map with or without HRH1-OE, in the presence of Tm/Tg (1 μM), relative to DMSO, 24 hours after exposure. Data are presented as means ± s.e.m, $n = 3$ independent replicates, with a two-tailed unpaired Student's t-test. **h** Relative XBP-1s-Luc ($n = 3$ independent replicates) activities in response to Tm/Tg at indicated concentrations, with or without HRH1-KD, 24 hours after exposure. **i** Immunoblot of HEK293T cells showing the protein levels of ER stress molecules in the IRE1α and ATF6 pathways. Quantification of relative protein levels. Data are presented as means ± s.e.m, $n = 3$ independent replicates, ***$P < 0.001$, **$P < 0.01$, *$P < 0.05$, ns: no significance, one-way ANOVA with Dunnett's multiple comparisons test. **j** RT-PCR showing the mRNA levels of un-spliced and spliced forms of XBP-1 (XBP-1u and XBP-1s). Source data are provided as a Source Data file.

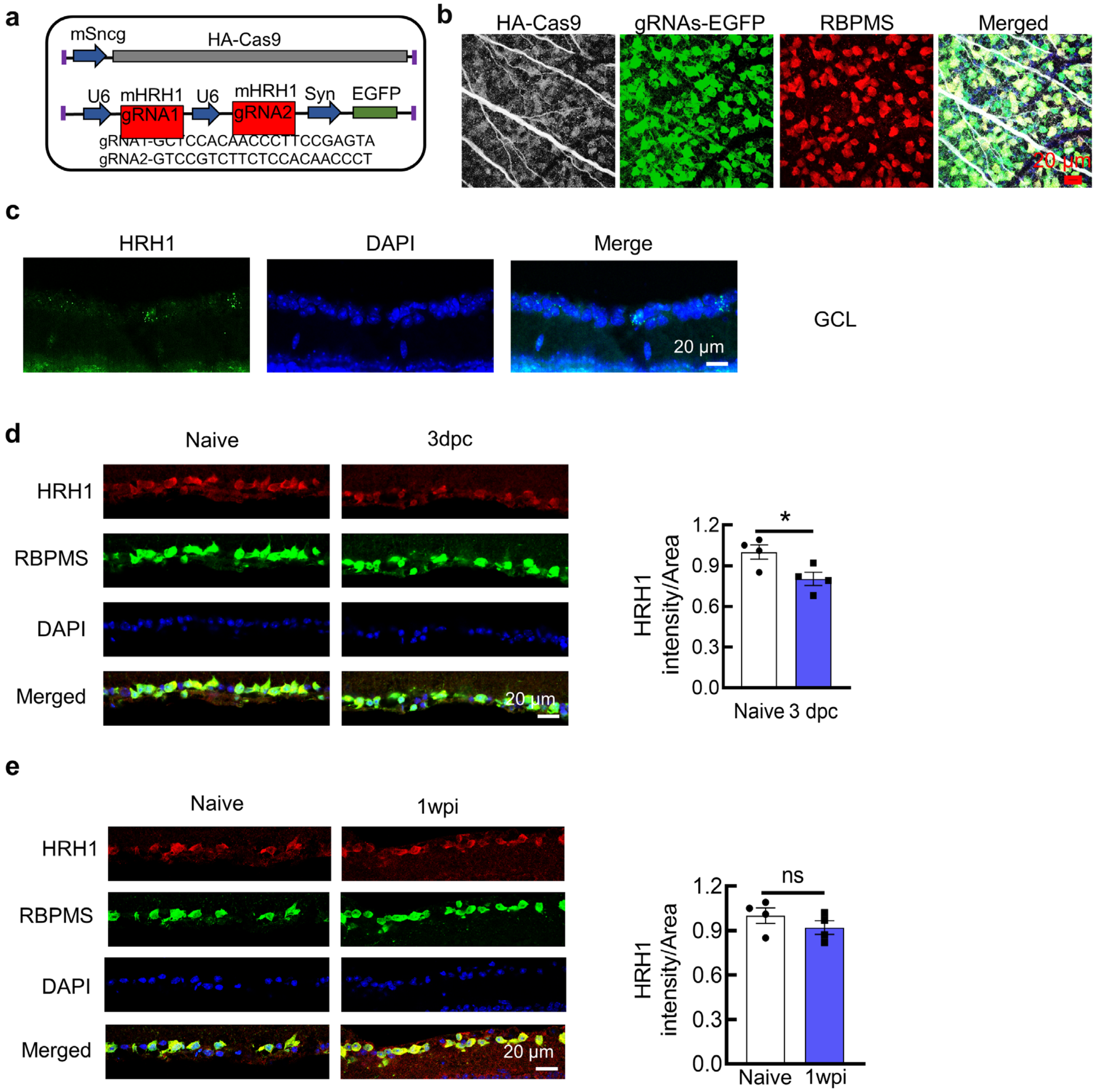

Supplementary Figure 7. AAV-mediated Cas9 and gRNA expression in RGCs and HRH1 levels in RGCs. a Schematic illustration of the AAV vectors expressing Cas9 under the mSncg promoter and a pair of gRNAs targeting mouse HRH1 (mHRH1). **b** Confocal images of flat-mounted retinas showing AAV-mediated *in vivo* expression of HA-tagged Cas9, gRNAs-EGFP and RBPMS+ RGCs. **c** Fluorescent *in situ* hybridization of mHRH1 mRNA in GCL of mouse retina sections. **d** Representative confocal images of retina section showing HRH1 protein levels in naïve and ONC (3dpc) mouse RGCs. **e** Representative confocal images of retina section

showing HRH1 protein levels in naïve and SOHU glaucoma (1wpi) mouse RGCs. Data are presented as means ± s.e.m, $n = 4$ mice, *$P < 0.05$, ns: no significance, with a two-tailed unpaired Student's t-test. Source data are provided as a Source Data file.

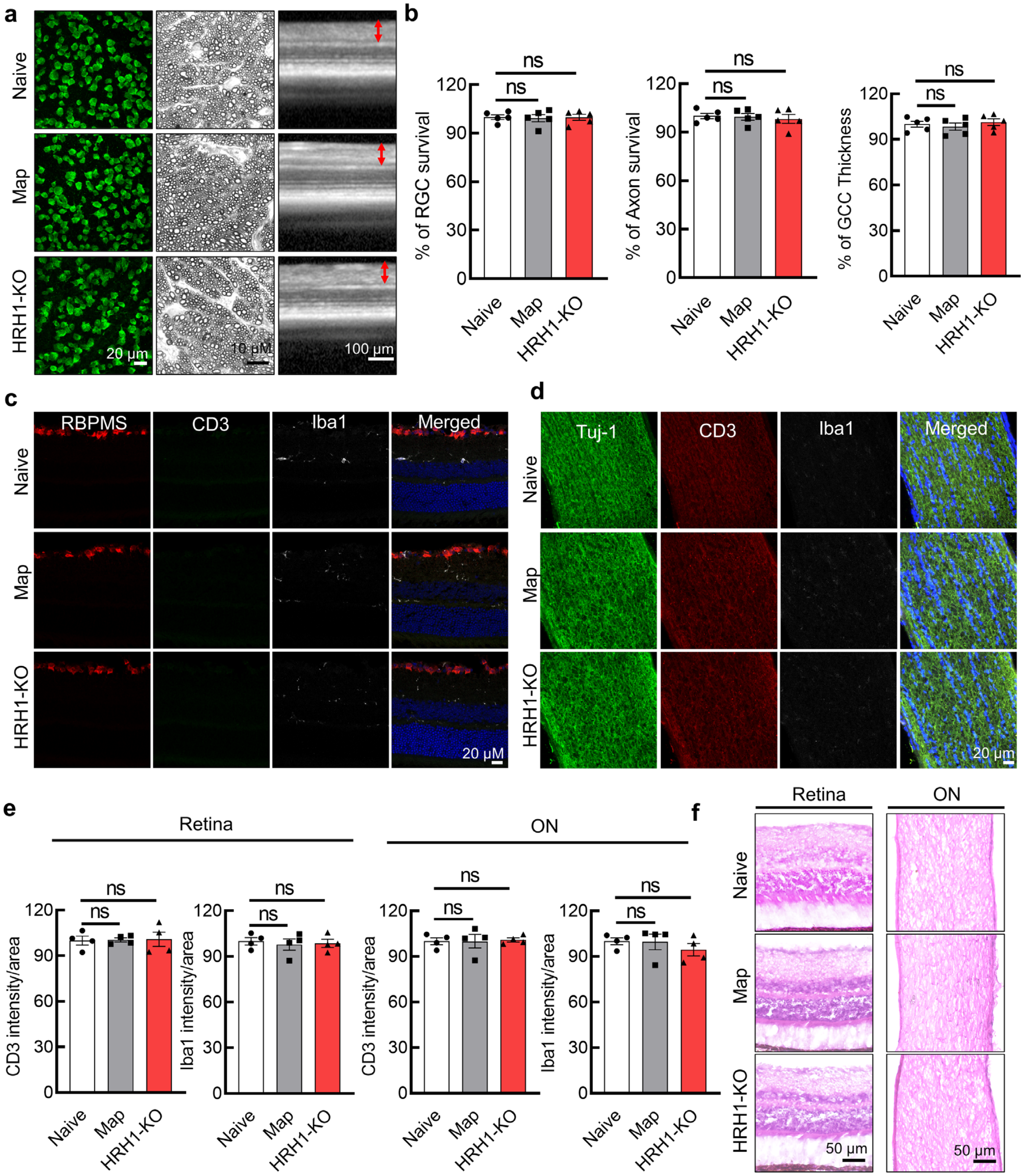

Supplementary Figure 8. No detectable long term safety issues with systemic Map administration or locally CRISPR-mediated HRH1 KD in RGCs. a Representative images of wholemount retina, ON section and in vivo OCT, after Map treatment for 1 month or AAV-

mediated CRISPR KD of HRH1 for 3 months. **b** Quantification of survival of RGC somata and axons and GCC thickness, represented as percentage of tested eyes compared to the sham contralateral control eyes. $n = 5$ mice. All data are presented as means ± s.e.m, ns: no significance, one-way ANOVA with Dunnett's multiple comparisons test. **c** Representative images of retina sections labeled with markers for RGC (RBPMS), T cell (CD3) and macrophage (Iba1). **d** Representative images of ON longitudinal sections labeled with axon marker (Tuj1), and cell markers for T cell (CD3) and macrophage (Iba1). **e** Quantification of inflammatory cell infiltration. $n = 4$ independent replicates. All the data are presented as means ± s.e.m, ns: no significance, one-way ANOVA with Dunnett's multiple comparisons test. **f** H&E staining of retina and ON sections after Map treatment for 1 month or AAV-mediated CRISPR KD of HRH1 for 3 months. Source data are provided as a Source Data file.

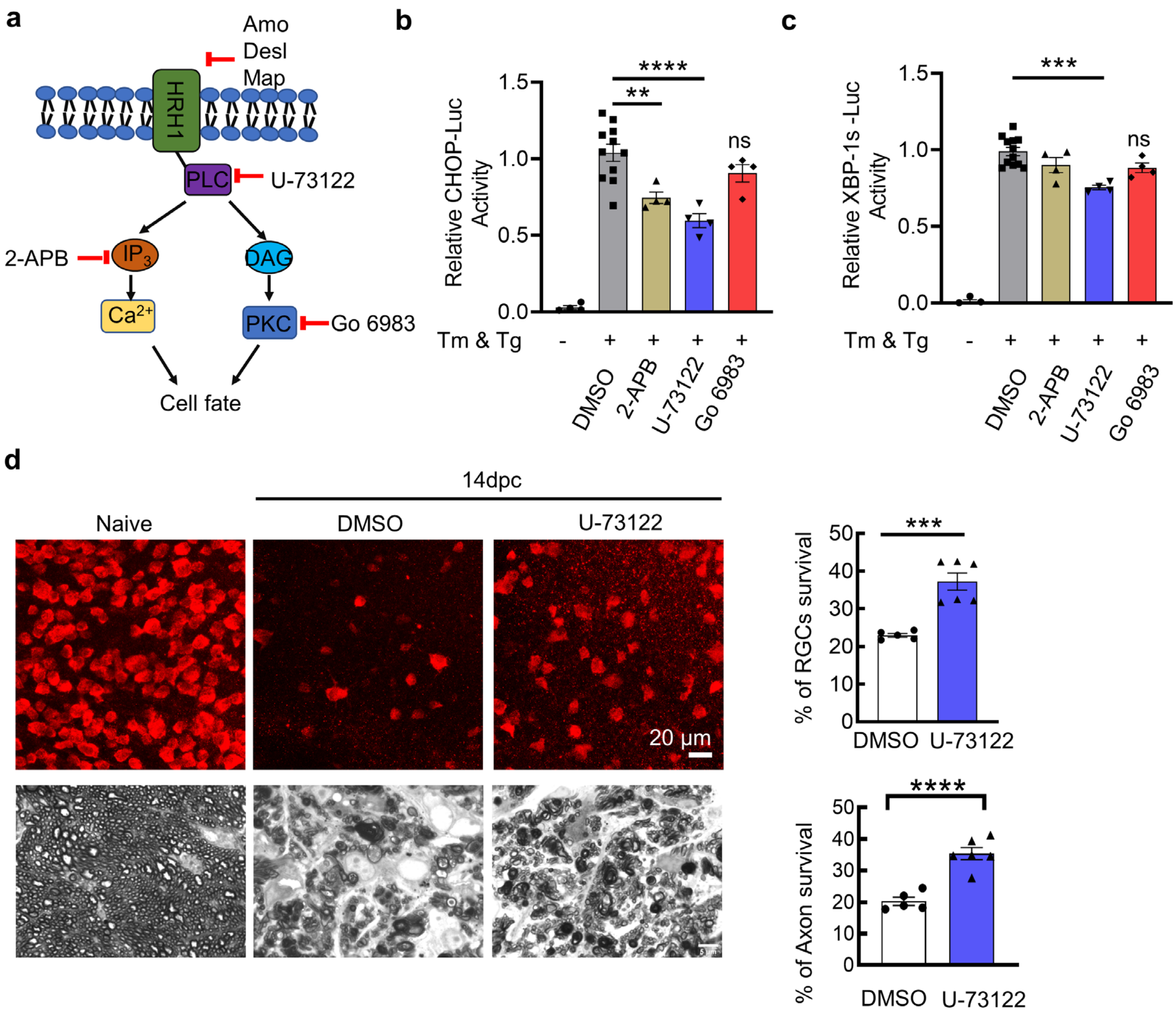

Supplementary Figure 9. ER stress modulation and neuroprotection with pharmacological inhibitors of downstream HRH1 signaling. a Schematic depicting the HRH1 signaling molecules and corresponding chemical inhibitors. (**b, c**) ER stress reporter assays with CHOP-Luc (**b**) and XBP1-Luc cells (**c**) in the presence of Tm&Tg with or without U-73122, 2-AP and Go 6983 treatments. *n* = 4 independent replicates for Ctr, 2-Ap, U73122 and GO6983; n = 11 independent replicates for DMSO; mean ± s.e.m; ****P* < 0.001, ***P* < 0.01, **P* < 0.05, ns: no significance, one-way ANOVA with Dunnett's multiple comparisons test. (**d**) Upper panel: representative confocal images of the wholemount retinas, showing surviving RBPMS+ (Red)

RGCs at 14dpc; Lower panel: representative light microscope images of semi-thin transverse sections of ON with PPD staining at 14dpc. Quantification of surviving RGC somata in wholemount retinas and axons in ON sections at 14dpc, represented as percentage of ONC eyes compared to the sham contralateral control eyes. $n = 6$ mice; data are presented as mean ± s.e.m, ***$P < 0.001$, ****$P < 0.0001$, with a two-tailed unpaired Student's t test. Source data are provided as a Source Data file.

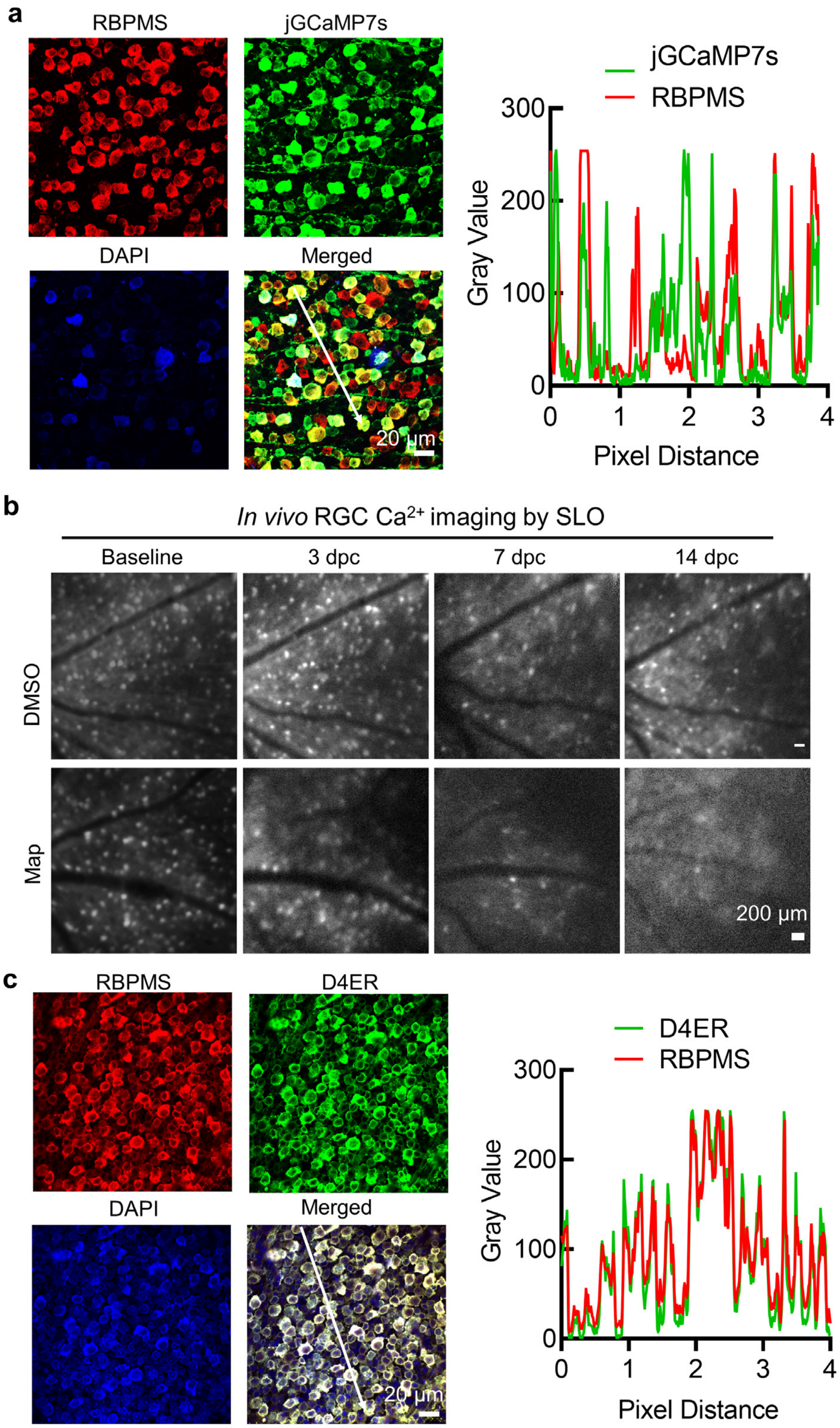

Supplementary Figure 10. AAV-mSncg promoter-mediated jGCaMP7s and D4ER expression in mouse RGCs *in vivo*. a Representative confocal images of wholemount retina

expressing jGCaMP7s 2 weeks after AAV-mSncg-jGCaMP7s intravitreal injection and colocalization of RGC marker RBPMS with jGCaMP7s analyzed by Image J. **b** *In vivo* retina imaging by SLO in living animals expressing jGCaMP7s in RGCs showing cytoplasmic Ca^{2+} influx in RGCs induced by ONC. **c** Representative confocal images of wholemount retina expressing D4ER 2 weeks after AAV-mSncg-D4ER intravitreal injection and colocalization of RGC marker RBPMS with D4ER analyzed by Image J.
